# Supplementary material for: The process of attrition in pre-medical studies: A large-scale analysis across 102 schools
Source: PLoS One. 2020 Dec 28;15(12):e0243546. doi: 10.1371/journal.pone.0243546 (PMC7769285; doi:10.1371/journal.pone.0243546)
Supplement: S1 Table — (DOCX) [file pone.0243546.s001.docx]

| **S1 Table** | | | | | | | | | | |
| --- | --- | --- | --- | --- | --- | --- | --- | --- | --- | --- |
| **Variable Intercorrelations Using Sub-Sample Included in the Logistic Regression Model for Milestone 1** | | | | | | | | | | |
|  | **1** | **2** | **3** | **4** | **5** | **6** | **7** | **8** | **9** | **10** |
| 1. SATC | 1.00 |  |  |  |  |  |  |  |  |  |
| 2. HSGPA | .34 | 1.00 |  |  |  |  |  |  |  |  |
| 3. SES | .39 | .09 | 1.00 |  |  |  |  |  |  |  |
| 4. Grades1 | .40 | .31 | .19 | 1.00 |  |  |  |  |  |  |
| 5. Grades12 | .40 | .32 | .19 | .95 | 1.00 |  |  |  |  |  |
| 6. OChem GR | .30 | .26 | .16 | .60 | .66 | 1.00 |  |  |  |  |
| 7. Milestone1 | .16 | .14 | .08 | .25 | .25 | .14 | 1.00 |  |  |  |
| 8. Milestone2 | .13 | .12 | .05 | .25 | .25 | .14 | .71 | 1.00 |  |  |
| 9. Milestone3 | .15 | .13 | .06 | .23 | .23 | .14 | .61 | .85 | 1.00 |  |
| 10. Milestone4 | .16 | .12 | .07 | .23 | .23 | .16 | .55 | .69 | .82 | 1.00 |
| *Note*. SATC = SAT composite, HSGPA = self-reported high school GPA, SES = socioeconomic status, Grade1 = average grades of first-semester general chemistry, biology, and physics, Grades12 = average grades of first- and second-semester general chemistry, biology, and physics, OChem GR = grade in first-semester organic chemistry, Milestone1 = whether students took a first semester of general chemistry, biology, and physics, Milestone2 = whether students who completed Milestone1 took a second semester of general chemistry, biology, and physics, Milestone3 = whether students who completed Milestone2 took a first semester of organic chemistry, Milestone4 = whether students who completed Milestone3 took a second semester of organic chemistry or a semester of biochemistry (full fulfillment). | | | | | | | | | | |
