# Supplementary material for: The process of attrition in pre-medical studies: A large-scale analysis across 102 schools
Source: PLoS One. 2020 Dec 28;15(12):e0243546. doi: 10.1371/journal.pone.0243546 (PMC7769285; doi:10.1371/journal.pone.0243546)
Supplement: S2 Table — (DOCX) [file pone.0243546.s002.docx]

| **S2 Table** | | | | | | | | | |
| --- | --- | --- | --- | --- | --- | --- | --- | --- | --- |
| ***Variable Intercorrelations Using Sub-Sample Included in the Logistic Regression Model for Milestone 2*** | | | | | | | | | |
|  | **1** | **2** | **3** | **4** | **5** | **6** | **7** | **8** | **9** |
| 1. SATC | 1.00 |  |  |  |  |  |  |  |  |
| 2. HSGPA | .28 | 1.00 |  |  |  |  |  |  |  |
| 3. SES | .39 | .07 | 1.00 |  |  |  |  |  |  |
| 4. Grades1 | .40 | .31 | .17 | 1.00 |  |  |  |  |  |
| 5. Grades12 | .39 | .32 | .17 | .95 | 1.00 |  |  |  |  |
| 6. OChem GR | .27 | .25 | .12 | .62 | .67 | 1.00 |  |  |  |
| 7. Milestone2 | .04 | .04 | .00 | .19 | .19 | .09 | 1.00 |  |  |
| 8. Milestone3 | .11 | .09 | .02 | .20 | .19 | .09 | .75 | 1.00 |  |
| 9. Milestone4 | .14 | .10 | .05 | .22 | .21 | .13 | .50 | .73 | 1.00 |
| *Note*. SATC = SAT composite, HSGPA = self-reported high school GPA, SES = socioeconomic status, Grade1 = average grades of first-semester general chemistry, biology, and physics, Grades12 = average grades of first- and second-semester general chemistry, biology, and physics, OChem GR = grade in first-semester organic chemistry, Milestone2 = whether students who completed Milestone1 took a second semester of general chemistry, biology, and physics, Milestone3 = whether students who completed Milestone2 took a first semester of organic chemistry, Milestone4 = whether students who completed Milestone3 took a second semester of organic chemistry or a semester of biochemistry (full fulfillment). | | | | | | | | | |
