# Supplementary material for: The process of attrition in pre-medical studies: A large-scale analysis across 102 schools
Source: PLoS One. 2020 Dec 28;15(12):e0243546. doi: 10.1371/journal.pone.0243546 (PMC7769285; doi:10.1371/journal.pone.0243546)
Supplement: Attachment — Submitted filename: PONE-D-20-15864_R1 LNB.pdf [file pone.0243546.s006.pdf]

PLOS ONE

The process of attrition in pre-medical studies: A large-scale analysis across 102 schools

--Manuscript Draft--

Manuscript Number:	PONE-D-20-15864R1
Article Type:	Research Article
Full Title:	The process of attrition in pre-medical studies: A large-scale analysis across 102 schools
Short Title:	Attrition in pre-medical studies
Corresponding Author:	Charlene Zhang<br>University of Minnesota<br>Minneapoilis, Minnesota UNITED STATES
Keywords:	pre-medical studies; Attrition; medical education; coursework; college performance
Abstract:	The important but difficult choice of vocational trajectory often takes place in college, beginning with majoring in a subject and taking relevant coursework. Of all possible disciplines, pre-medical studies are often not a formally defined major but pursued by a substantial proportion of the college population. Understanding students' experiences with pre-med coursework is valuable and understudied, as most research on medical education focuses on the later medical school and residency. We examined the pattern and predictors of attrition at various milestones along the pre-med coursework track during college. Using a College Board dataset, we analyzed a sample of 15,442 students spanning 102 institutions who began their post-secondary education in years between 2006 and 2009. We examined whether students fulfilled the required coursework to remain eligible for medical schools at several milestones: 1) one semester of general chemistry, biology, physics, 2) two semesters of general chemistry, biology, physics, 3) one semester of organic chemistry, and 4) either the second semester of organic chemistry or one semester of biochemistry, and predictors of persistence at each milestone. Only 16.5% of students who intended to major in pre-med graduate college with the required coursework for medical schools. Attrition rates are highest initially but drop as students take more advanced courses. Predictors of persistence include academic preparedness before college (e.g., SAT scores, high school GPA) and college performance (e.g., grades in pre-med courses). Students who perform better academically both in high school and in college courses are more likely to remain eligible for medical school.
Order of Authors:	Charlene Zhang
	Nathan R Kuncel
	Paul R Sackett
Opposed Reviewers:	
Response to Reviewers:	Your paper received comments from four reviewers. They all found the theme of the paper important. However, they shared several concerns with the paper. I am particularly concerned the missing issues in the paper as these issues if addressed could improved the quality of the paper's message.<br><br>Dear Dr. Borrell,<br><br>Thank you for initiating reviews on our paper "The process of attrition in pre-medical studies: A large-scale analysis across 102 school" and giving us the opportunity to revise and resubmit. We appreciated the reviews and constructive comments provided by the reviewers and yourself.<br><br>It was encouraging to see that reviewers shared our view in the importance of this work. We had a unique opportunity to quantitatively examined the process through which undergraduate students fulfill prerequisite coursework for medical schools using a large multi-cohort, multi-institution sample. Given the popularity of the informal pre-med major and little prior work focusing on attrition in the early pre-medical study

Powered by Editorial Manager® and ProduXion Manager® from Aries Systems Corporation

stage, we felt that this work is valuable in adding to the current higher education literature.

In our response to reviewer comments, we have made effort to address every reviewer comment point-by-point and to incorporate the feedback into our revised manuscript. Notably, we substantially restructured our Table 1 to describe the key fulfillment rates in a way that is clearer and also include rates at the intersection of gender and race and at various fulfillment milestones. We also tested additional models that included interaction terms between the various predictors. The results were not tabled given the large number of complex models, but we described key findings. Lastly, we added discussions of the recent shift to holistic evaluation of applicants in medical education and its implications for the ongoing relevance of our results.

A minor detail to note is that while "degree goal" was a predictor included in our original submission because it was a variable that existed in the archival data given to us, and has often been found to be predictive of academic performance in past research. However, upon reexamination, we deemed it irrelevant to the current work. We define our total sample as individuals who have intention of becoming physicians, therefore their "degree goal," or the highest degree they hope to pursue, had very little variance. We excluded it in the revised manuscript, but none of the reported findings changed.

We believe that these edits, along with other minor revisions, have significantly improved our manuscript. Thank you again for your consideration.

Reviewer #1: This manuscript provides a valuable analysis of the academic trajectories of students who enter college with an interest in pursuing premedical studies, and subsequently to attend medical school. Using a large data set provided by the College Board, the authors were able to identify students who had expressed the interest in premedical studies based on students' response to a question administered at the time students took the SAT exam. As the authors indicate, more than 15,000 students indicated the intent to pursue premedical studies once they entered college.

The metric the authors utilize to gauge students' continued level of interest in being premed is the extent to which students complete the science courses that have traditionally been required by medical schools for entry. They analyze the successful completion of these courses in a stepwise manner, as described in the Methods section. Of the students included in the data set, 16.5% completed the full sequence of courses identified by the authors by the time they had completed their undergraduate education. The authors then did an analysis of the academic and demographic factors associated with full completion of the course sequence. They identified gender, race/ethnicity, and prior academic record as among the factors associated with completion of the full course sequence.

This manuscript provides valuable information, potentially useful to those who advise undergraduates in career options. The data set the authors use is unique in its size and the variables it contains. As such, the manuscript adds value to our knowledge of the factors associated with persistence in interest in premedical studies, which is an important factor in the eventual diversity of the physician workforce.
We appreciate the positive feedback on the value of this work. We agree with the reviewer that this dataset provides a unique opportunity to examine the academic trajectories of pre-medical students.
The manuscript reports data on students who entered college between 2006 and 2009. Most of these students would have completed their undergraduate education by 2013-2014. As such, the analysis has missed some fundamental changes that have taken place in the premedical experience. As a result, some of their assumptions and findings may need re-examination by the authors. As one example, the authors state in the Abstract, "Attrition rates are highest initially but drop as students take more relevant courses." The issue is not the relevance of the courses. The issue is how advanced the courses are. The AAMC has done research indicating that inorganic chemistry courses have more relevance to the practice of medicine than do organic chemistry courses. Organic chemistry courses are more advanced than inorganic chemistry courses, but they are not more "relevant".
We have changed "relevant" to "advanced" in the abstract as we agree that later, more advanced courses are not necessarily more relevant that earlier courses. We use the

description “relevant” at several other places throughout the manuscript to refer to all courses used to operationalize medical school eligibility that are common prerequisites.

A second thing the authors miss in their discussion is the fundamental shift that has been taking place in the medical school admission process, with the shift to holistic review. In April 2013, Witzburg and Sondheimer published an article in the New England Journal of Medicine titled, “Holistic Review — Shaping the Medical Profession One Applicant at a Time.” Under holistic review, as described by the AAMC, a student’s grades in the premedical science courses, while still relevant, are only one of a series of factors used in selecting candidates for medical school. Consistent with the holistic review model, a growing number of medical schools no longer have a specific list of prerequisite courses required for admission. For example, the Perelman School of Medicine at the University of Pennsylvania reports on its Admissions website, “Admissions competencies are not based on specific courses, but rather on the cumulative achievement of knowledge and skills needed to become a physician.” (https://www.med.upenn.edu/admissions/admissions.html)
Similarly, the Admissions webpage of the Stanford University School of Medicine states explicitly, “Stanford Medicine does not have specific course requirements, but recommends appropriate preparation for the study of medicine.” (http://med.stanford.edu/md-admissions/how-to-apply/academic-requirements.html)
Those medical schools that have dropped their explicit course requirements are instead relying on the newly formatted MCAT to provide a metric of scientific and behavioral competencies. Accordingly, in order for this manuscript to sustain its relevance to the current premedical experience, the authors should include a full discussion of the changes that have taken place since their study cohort graduated from college.

We thank the reviewer for pointing out the substantial shift in the evaluation process of medical school applicants. Given when our data was collected and how relatively uniform the prerequisite requirements were across medical schools during that time period, we were able to operationalize medical school eligibility using coursework. As the emphasis on prerequisites relaxes, this approach will become no longer reliable. Per the reviewer’s suggestion, we add some language in the limitations and future directions section to highlight this issue.
There is another important factor the authors have left out of their analysis and discussion. While most students who enter college with an intent to pursue premedical studies will complete the sequence of science courses at their undergraduate institution, a substantial number will elect to take either no science courses or some science courses at their undergraduate institution, while completing the full list of traditional science courses as part of a postbaccalaureate premedical program. The AAMC currently identifies 267 programs nationally (https://apps.aamc.org/postbac/#/index) that offer students the opportunity to complete their premedical course sequence after they have graduated from their undergraduate institution. Most of these programs have been active for more than a decade, and thus were available to the students included in the authors’ data set. The authors are unable to identify which of the students elected to take only some courses as an undergraduate, while completing the course sequence in a past-bac program.

It is certainly true that our approach of identifying students with intention in studying medicine and operationalizing their continued persistence is not perfect. As the first of the limitations we pointed out, by focusing on coursework, we inevitably include those that have the required coursework for most medical schools but nevertheless lost their intention to study medicine along the way. We thank the reviewer for pointing out another group considered as noise, namely those that do not complete the required coursework during their undergraduate years, but eventually do in a postbaccalaureate program. We added language to reflect this limitation.
I should point out that, due to poor quality graphics of Figure 1 in the manuscript, I was unable to decipher the figure. The text and images were so faint that I could not read them. I would ask the authors to revise the figure with improved graphics.
The figure has been regenerated to be higher-quality.

As one final issue, I suggest the authors include in their discussion the results of a paper that was published this month that reported on the association between grades

Powered by Editorial Manager® and ProduXion Manager® from Aries Systems Corporation

in undergraduate chemistry classes and persistence in STEM majors:
R. B. Harris et al. Reducing achievement gaps in undergraduate general chemistry could lift underrepresented students into a “hyperpersistent zone”. Science Advances. 10 Jun 2020:
Vol. 6, no. 24, eaaz5687 DOI: 10.1126/sciadv.aaz5687
We have added reference of the study. We found the key results of interaction between race and grades for predicting subsequent persistence intriguing. Therefore, we also tested models additional to the ones reported in Table 3 that included various interactions between demographic dummies and continuous predictors. Because this yielded 32 additional models that contained largely redundant and statistically insignificant results, they are not tabled and presented. However, we added a paragraph at the end of the Results section describing the significant interactions.
Reviewer #2: 0. This paper is important for empirically investigating the 'pre-med pipeline,' where many students enter into pre-med (an unspecified 'major' at many schools, including mine) -- and many drop out. Who are these students, and how does dropout unfold over time? That is the focus of the work, which should be of great interest to a wide range of stakeholders: e.g., universities, students and parents, STEM researchers, and medical educators and institutions. Authors have large-scale transcript data across 102 institutions; they have students' major intentions prior to college enrollment -- an impressive data set to answer these questions.

1. A passing grade (.7 GPA or higher) was used as a screen for eligibility to continue taking pre-med courses, but authors might clarify how this was used (being eligible is necessary but not sufficient for taking the next course). Related to this (but entirely optional), authors might indicate % failure (and didn't retake) of initial courses by demographics as one barrier to entry; and/or investigating course grades more generally might predict staying in pre-med.
As we operationalized medical school eligibility as the fulfillment of various relevant courses, the passing grade was merely used as a minimum standard for which a course can be counted toward achievement. For example, the first milestone requires the completion of one semester of general chemistry, biology, and physics with a passing grade. We revised the language of the four milestones to make this clearer.
2. p. 13 - One can easily appreciate how SAT, HSGPA, and CGPA would be higher for course-fulfillers, to the extent non-fulfilling is driven by obtaining low grades (or failures) in pre-med courses; this would suggest examining or at least suggesting course grades as an explanatory mechanism (see previous point). Regarding SES differences, could they be higher if college dropouts were included (those who couldn't afford to continue at all, let alone in pre-med)? Dropout might be a significant problem for pre-med and STEM majors, and for URM students, but the current sample does not include this group (maybe include this point somewhere in the discussion section, perhaps with some data on % dropout in the College Board student data).

We agree with the reviewer about the merit of including course grades as a predictor. Our current logistic regression models include average prior relevant course grade as a predictor when possible. We also tested but did not report results of models that included individual course grades separately, as the models currently reported are more parsimonious and yielded similar results.
Unfortunately, the data we were given do not let us identify students that drop out. By examining coursework data, we are able to identify individuals that have coursework up to some point during their undergraduate years, but do not complete all coursework. However, we have no means of distinguishing between those that truly dropped out and those that transferred to a different institution.

3. Table 3 and related text - (a) interpretations of odds ratios need to be refined (they are with respect to the reference group of white females, not overall; they also need to take other predictors into account); (b) odds ratios can be usefully compared against base rate %s from Table 2 and the raw data (e.g., plug in values into the equation to get predicted probabilities, to compare against base rate%s); (c) the two previous points get at whether the pseudo-R2 values are practically useful; but in addition to this, I'd recommend a formal statistical comparison between adjacent models (e.g., compare AIC values).
We thank the reviewer for urging us to report regression findings with more accurate language. We have revised the results section to specify the reference group of the dummy variables as well as the interpretation of coefficients when controlling for all

Powered by Editorial Manager® and ProduXion Manager® from Aries Systems Corporation

other predictors.
We also agree that odds ratios can be unintuitive and difficult to interpret, and describing the effects in terms of predicted probabilities can facilitate interpretation. Therefore, we have revised the results section by translating some of the key coefficients and odds ratios into differences in likelihood.
We have also added AIC values of the regression models that we tested in Table 3.

4. A bit surprisingly, no institutional differences were provided -- even just adding something such as the distribution of %fulfill by institution would be interesting to readers (and this could be relatively anonymous, given 102 institutions).

We agree with the reviewer that some attention should be paid to institutional differences. The sample sizes per institution varied substantially in the current data, ranging from 2 to 1,475. Therefore, proportions of students that fulfilled coursework milestones in institutions for which we only had sample sizes in the single digits could lead to misleading conclusions. However, we provide an added paragraph near the beginning of the results section with the mean and standard deviation of fulfillment proportion across institutions. We hope this sheds some light on the fulfillment distributions.

Side notes:
5. Table 1 - proportion row/column information was useful, but the labeling was a little confusing
We agree that the original Table 1 could benefit from clearer labels. In addition, we received suggestions from other reviewers to expand this table to also look at the fulfillment breakdown at the intersection of gender and race, as well as to look at fulfillment proportions at the intermediate milestones. In an effort to take into consideration all of these suggestions, we completely restructured our Table 1 to provide the sample size and percentage of individuals that fulfilled each of the four coursework milestones for the entire sample, by gender, by race, and by gender and race. By doing so, we focus the purpose of the table on displaying the proportion who remain med-school-eligible at each stage, and how the proportions compare across demographic groups. We think that this significantly improves the interpretability and readability of the table.

6. Table 2 - (a) the # of courses help define the fulfill/non-fulfill categories so the d-values may not be surprising (if you include them, also check out the distributions of N, as maybe there's positive skew and you consider a more robust d-value involving medians); (c) format the rows a little more descriptively (headers? better labels?)
The reviewer is correct in pointing out that the number of courses taken are quite different between fulfillers and non-fulfillers by definition and they are skewed. We have revised the table to show Cliff's delta rather than Cohen's d for those variables. Row names have been revised to be more descriptive and sub-headings have been added better describe the variables.
Reviewer #3: This study examines a key outcome in the medical education track, and brings to light quantitative research findings that should provide a valuable contribution to the literature for policy-makers and admissions officers alike. The submission is clearly structured, and the analyses are appropriate to the research question, which complements existing work with a more quantitative and large scale data-based approach. I only have a handful of comments.

1) The limitations mentioned in the study are appropriate to mention, but I would have liked to also see further discussion on the relevance of data that is between 11 and 14 years old to the world of medical education today. How stable are the trends that are noted in comparison to the trends of today? Beyond noting the structural changes to MCAT, are there other factors that would lead to caution in drawing strong conclusions from this data, such as the final sentence of the conclusion?
We thank the reviewer for the thoughtful comment. There certainly are additional factors that might influence the generalizability and relevance of the findings. A major one is the endorsement of "holistic review" by medical applicant evaluation and the shift from primary emphasis on academic performance to a consideration of all aspects of applicants' experiences and potential. We added some language in the limitations section explaining this shift in the past decade and its implications.
2) When comparing fulfillers and non-fulfillers of medical school prerequisites, the

Powered by Editorial Manager® and ProduXion Manager® from Aries Systems Corporation

paper highlights and contextualizes the larger proportion of male fulfillers, but is there a clarifying sentence that can also be added regarding meaningful differences with regards to race/ethnicity?
We have revised the description of those results to reflect the differences.
3) Examining subgroup differences in attrition is a key valuable component of the study, and to that end the paper would be improved by some examination or at the very least mention of interactive effects between race/ethnicity and gender and attrition. For example, the report Altering the Course: Black Males in Medicine highlights the percentage of black male medical school applicants as the lowest among any subgroup. Would conclusions such as ""Lastly, African American students did not differ from White students in their likelihood of persistence at any point." (p 19) differ when differentiating between black men and black women?
We think that the reviewer brings up an important issue regarding the interaction between gender and race for likelihood of persistence. We tried to address this issue in two different ways. First, we expanded and restructured Table 1 to describe not only fulfillment rates by gender and by race separately, but also at the intersection of race and gender. This allows us to compare both the number of individuals who declare some intention to pursue medicine as well as persistence patterns over time across demographic groups (e.g., between Black men and Black women). In addition to these descriptives, we also tested the same four models whose results are displayed in Table 3 when including the interaction terms between gender and race dummy variables. Because the inclusion of interaction terms would add an additional table that is similar to but much larger than Table 3 and more cumbersome to interpret, and most of the coefficients are statistically not significant, we did not table those results but describe the significant effects at the end of the Results section.
Reviewer #4: This study investigates the completion rates of students completing the sequence of undergraduate natural science coursework prerequisite for entry to medical school in the U.S. using a large-scale, multi-institution dataset. It examines the associations of multiple sociodemographic, pre-college achievement, career aspiration, and college-level achievement measures in relation to students' completion of four milestone events defined by natural science coursework from the first semester through advanced organic or biochemistry courses.

The study showed that, of those who reported intending to pursue a medical degree when they took the SAT in high school, only 39% completed the first in a sequence of courses that together provide the foundation needed to be ready for medical school. Completion rates were progressively higher for the (likely) increasingly select group of students achieving each of the three subsequent milestones of coursework. In the end, the overall completion rate of all four milestones was 16.5%. Observed proportions completing the full sequence were higher for males and Asian students, and lower for females and students from races/ethnicities underrepresented in medicine (i.e., American Indian, Black, and Hispanic).

High school GPA, SAT scores, SES, Gender (Male), and Race (Asian) were associated with greater likelihoods of completing the first set of milestone courses. Gender (Male), Race (Hispanic), and college grades in the first milestone courses were associated with greater likelihoods of completing the second milestone courses, which included the second semester courses of the subjects comprising the first milestone. SAT scores and high school GPA were associated with greater likelihoods of completing the third milestone courses, and race (Hispanic) with a lower likelihood. Finally, gender (Male) and intention to pursue medical school were associated with greater likelihoods of completing the fourth milestone courses, while SAT scores were associated with a lower likelihood of completing the fourth milestone courses.

Although this study has the potential to enhance our understanding of the points at which those interested in the medical profession drop out, the paper fails to make a strong case for it.

1. The authors should consider rewriting the introduction and discussion with a clearer focus and relevant citations, considering the following as suggestions.
• Integrating studies about the factors that contribute to a lack of diversity in STEM or medicine that occur during or before college with the present study, including those referenced in the current about "the leaky pipeline." References 4, and 11-19 seem germane to the study's focus.

	• Addressing, alongside things like gender norms that might affect persistence, comparable treatment of the lower rates of completion for those from racial/ethnic backgrounds underrepresented in medicine who, more often have lower-quality middle and high school education. Addressing the role that high school preparation might play in students first-semester performance in natural science coursework and beyond would strengthen the paper.<br>• Removing or clarifying the relevance of literature on burnout/risk of attrition in medical school (references 3, and 5-9), and references 3, 10, and 20, which about attrition in the medical school in the U.K., which differs substantially from medical school in the U.S. The authors also should consider eliminating references about attrition in U.S. medical school (given that 95% of medical students graduate within five years) or make a stronger case for their relevance.<br><br>We have revised the introduction and discussion following some of the reviewers' suggestions, including removing references of studies of medical school in UK, removing review of the literature on attrition in medical school, and adding some language on systemic underrepresentation of ethnic minority groups in medicine.<br><br>2. With a clearer focus, the authors might present their results differently to highlight important findings. For example, Table 1 shows that Black and Hispanic students completed all four milestones at lower rates than White or Asian students. It might be important, given the research questions, to know at which milestone(s) they did not progress, in addition to showing their prevalence at the two end points. Similarly, it was confusing on page 19 to read that African American students did not differ from White students in their likelihood of persistence at any point, even though only 9% of Black students completed all four milestones, compared to 16% of White students. The authors should address how the observed and predicted rates of completion lead to different interpretations.<br><br>3. Similar suggestions would improve the discussion of the paper's findings.<br>We have expanded and restructured Table 1 and present fulfillment rates by racial subgroups at various milestones, following the reviewer's suggestions. We agree that by doing so, we gain a more complete understanding of the process of attrition.<br>We thank the reviewer for pointing out the confusion in presenting proportion of fulfillment by group and including those subgroup dummy variables in the regression models. For example, the African American group had the overall lowest proportion of overall eligibility based on their coursework. However, this difference seems to go away in the predictive models once the other predictors (e.g., SES, SAT scores, and grades) are controlled. We have revised the language in the Results and Discussion sections to clarify this.<br><br>4. Finally, the conclusions are unfounded and should be rewritten.<br><br>5. Other minor suggestions include:<br>• addressing how the increasingly select sample of students might affect the results of the logistic regression analyses. For example, SES, SAT scores, and high school GPA may be restricted as the sample reduces from more than 13,000 to about 2,500 students.<br><br>• Replacing outdated references with more recent research (e.g., 18) and confirming the appropriateness of journals cited (e.g., 9, which is missing the Journal Name "Journal of Unschooling and Alternative Learning").<br><br>• Clarifying the relevance or eliminating the text describing challenges identifying premed majors. The study documented the process for identifying courses prerequisite for medical school (and that are well described on each medical school's website).<br>References have been edited and the Conclusion section was rewritten.
**Additional Information:**	
**Question**	**Response**
**Financial Disclosure**<br><br>Enter a financial disclosure statement that	This research was supported by a grant from the College Board to Paul R. Sackett and Nathan R. Kuncel. Paul R. Sackett serves as a consultant to the College Board. This relationship has been reviewed and managed by the University of Minnesota in

describes the sources of funding for the work included in this submission. Review the submission guidelines for detailed requirements. View published research articles from *PLOS ONE* for specific examples.<br><br>This statement is required for submission and **will appear in the published article** if the submission is accepted. Please make sure it is accurate.<br><br>**Unfunded studies**<br>Enter: *The author(s) received no specific funding for this work.*<br><br>**Funded studies**<br>Enter a statement with the following details:<br>• Initials of the authors who received each award<br>• Grant numbers awarded to each author<br>• The full name of each funder<br>• URL of each funder website<br>• Did the sponsors or funders play any role in the study design, data collection and analysis, decision to publish, or preparation of the manuscript?<br>• **NO** - Include this sentence at the end of your statement: *The funders had no role in study design, data collection and analysis, decision to publish, or preparation of the manuscript.*<br>• **YES** - Specify the role(s) played.<br><br>* typeset	accordance with its conflict of interest policies. This research is derived from data provided by the College Board. Copyright 2006–2011 The College Board. www.collegeboard.com<br><br>The funders had no role in study design, data collection and analysis, decision to publish, or preparation of the manuscript.
**Competing Interests**<br><br>Use the instructions below to enter a competing interest statement for this submission. On behalf of all authors, disclose any competing interests that could be perceived to bias this work—acknowledging all financial support and any other relevant financial or non-financial competing interests.<br><br>This statement **will appear in the published article** if the submission is accepted. Please make sure it is accurate. View published research articles from *PLOS ONE* for specific examples.	The authors have declared that no competing interests exist.

Powered by Editorial Manager® and ProduXion Manager® from Aries Systems Corporation

**NO authors have competing interests**<br><br>Enter: *The authors have declared that no competing interests exist.*<br><br>**Authors with competing interests**<br><br>Enter competing interest details beginning with this statement:<br><br>*I have read the journal's policy and the authors of this manuscript have the following competing interests: [insert competing interests here]*<br><br>* typeset	
**Ethics Statement**<br><br>Enter an ethics statement for this submission. This statement is required if the study involved:<br><br>• Human participants<br>• Human specimens or tissue<br>• Vertebrate animals or cephalopods<br>• Vertebrate embryos or tissues<br>• Field research<br><br>Write "N/A" if the submission does not require an ethics statement.<br><br>General guidance is provided below. Consult the submission guidelines for detailed instructions. **Make sure that all information entered here is included in the Methods section of the manuscript.**	N/A

**Format for specific study types**<br><br>**Human Subject Research (involving human participants and/or tissue)**<br>• Give the name of the institutional review board or ethics committee that approved the study<br>• Include the approval number and/or a statement indicating approval of this research<br>• Indicate the form of consent obtained (written/oral) or the reason that consent was not obtained (e.g. the data were analyzed anonymously)<br><br>**Animal Research (involving vertebrate animals, embryos or tissues)**<br>• Provide the name of the Institutional Animal Care and Use Committee (IACUC) or other relevant ethics board that reviewed the study protocol, and indicate whether they approved this research or granted a formal waiver of ethical approval<br>• Include an approval number if one was obtained<br>• If the study involved *non-human primates*, add *additional details* about animal welfare and steps taken to ameliorate suffering<br>• If anesthesia, euthanasia, or any kind of animal sacrifice is part of the study, include briefly which substances and/or methods were applied<br><br>**Field Research**<br><br>Include the following details if this study involves the collection of plant, animal, or other materials from a natural setting:<br>• Field permit number<br>• Name of the institution or relevant body that granted permission	
**Data Availability**<br><br>Authors are required to make all data underlying the findings described fully available, without restriction, and from the time of publication. PLOS allows rare exceptions to address legal and ethical concerns. See the PLOS Data Policy and FAQ for detailed information.	No - some restrictions will apply

A Data Availability Statement describing where the data can be found is required at submission. Your answers to this question constitute the Data Availability Statement and **will be published in the article**, if accepted.<br><br>**Important:** Stating 'data available on request from the author' is not sufficient. If your data are only available upon request, select 'No' for the first question and explain your exceptional situation in the text box.<br><br>Do the authors confirm that all data underlying the findings described in their manuscript are fully available without restriction?	
**Describe where the data may be found in full sentences. If you are copying our sample text, replace any instances of XXX with the appropriate details.**<br><br>• If the data are **held or will be held in a public repository**, include URLs, accession numbers or DOIs. If this information will only be available after acceptance, indicate this by ticking the box below. For example: *All XXX files are available from the XXX database (accession number(s) XXX, XXX.).*<br>• If the data are all contained **within the manuscript and/or Supporting Information files**, enter the following: *All relevant data are within the manuscript and its Supporting Information files.*<br>• If neither of these applies but you are able to provide **details of access elsewhere**, with or without limitations, please do so. For example:<br><br>*Data cannot be shared publicly because of [XXX]. Data are available from the XXX Institutional Data Access / Ethics Committee (contact via XXX) for researchers who meet the criteria for access to confidential data.*<br><br>*The data underlying the results presented in the study are available from (include the name of the third party*	The dataset used for this project is proprietary, owned and collected by a third-party, and cannot be freely posted online. We did not have any special access to the data that other researchers would not have. Researchers interested in the data for replication purposes only. May request access by contacting the College Board here<br>The College Board<br>250 Vesey Street<br>New York, NY 10281<br>212-713-8088<br>Or through the direct online portal here: http://research.collegeboard.org/data/request<br>Please reference the paper and authors in the request.

and contact information or URL).<br>• This text is appropriate if the data are owned by a third party and authors do not have permission to share the data.<br><br>* typeset	
Additional data availability information:	

Powered by Editorial Manager® and ProduXion Manager® from Aries Systems Corporation

PLOS ONE

Submission Date: October 16, 2020

Dear Editorial Board,

I am re-submitting our revised manuscript entitled, “The Process of Attrition in Pre-Medical Studies: A Large-Scale Analysis Across 102 Schools” (PONE-D-20-15864) co-authored by Charlene Zhang, Nathan R. Kuncel, and Paul R. Sackett. Thank you for initiating reviews on our paper and giving us the opportunity to revise and resubmit. We appreciated the reviews and constructive comments provided by the reviewers and yourself.

It was encouraging to see that reviewers shared our view in the importance of this work. We had a unique opportunity to quantitatively examined the process through which undergraduate students fulfill prerequisite coursework for medical schools using a large multi-cohort, multi-institution sample. Given the popularity of the informal pre-med major and little prior work focusing on attrition in the early pre-medical study stage, we felt that this work is valuable in adding to the current higher education literature.

The manuscript has not been published previously, and is not under review elsewhere. Each of the authors confirms that this manuscript has not been previously published and is not currently under consideration by any other journal. Additionally, all of the authors have approved the contents of this paper and have agreed to PLOS’s submission policies. Each named author has substantially contributed to conducting the underlying research and drafting this manuscript. Additionally, to the best of our knowledge, the named authors have no conflict of interest, financial or otherwise.

Data Availability Statement
The dataset used for this project is proprietary, owned and collected by a third-party, and cannot be freely posted online. We did not have any special access to the data that other researchers would not have. Researchers interested in the data for replication purposes only. May request access by contacting the College Board here
The College Board
250 Vesey Street
New York, NY 10281
212-713-8088
Or through the direct online portal here: http://research.collegeboard.org/data/request
Please reference the paper and authors in the request.

Sincerely,

Charlene Zhang (B.S.)—Corresponding Author
University of Minnesota
75 E River Rd #N496, Minneapolis, MN 55455
zhan5449@umn.edu
Tel: 612-839-2310

Nathan R. Kuncel (Ph.D.)
University of Minnesota
75 E River Rd #N218, Minneapolis, MN 55455
kunce001@umn.edu
Tel: 612-624-1651

Paul R. Sackett (Ph.D.)
University of Minnesota
75 E River Rd #N475, Minneapolis, MN 55455
psackett@umn.edu
Tel: 612-624-9842

Manuscript Click here to access/download;Manuscript;PLOS_manuscript_200525.docx

The process of attrition in pre-medical studies: A large-scale analysis across 102 schools

Short title: Attrition in pre-medical studies

Charlene Zhang (B.S.)—Corresponding Author
University of Minnesota
75 E River Rd #N496, Minneapolis, MN 55455
zhan5449@umn.edu
Tel: 612-839-2310

Nathan R. Kuncel (Ph.D.)
University of Minnesota
75 E River Rd #N218, Minneapolis, MN 55455
kunce001@umn.edu
Tel: 612-624-1651

Paul R. Sackett (Ph.D.)
University of Minnesota
75 E River Rd #N475, Minneapolis, MN 55455
psackett@umn.edu
Tel: 612-624-9842

Abstract

The important but difficult choice of vocational trajectory often takes place in college, beginning with majoring in a subject and taking relevant coursework. Of all possible disciplines, pre-medical studies are often not a formally defined major but pursued by a substantial proportion of the college population. Understanding students' experiences with pre-med coursework is valuable and understudied, as most research on medical education focuses on the later medical school and residency. We examined the pattern and predictors of attrition at various milestones along the pre-med coursework track during college. Using a College Board dataset, we analyzed a sample of 15,442 students spanning 102 institutions who began their post-secondary education in years between 2006 and 2009. We examined whether students fulfilled the required coursework to remain eligible for medical schools at several milestones: 1) one semester of general chemistry, biology, physics, 2) two semesters of general chemistry, biology, physics, 3) one semester of organic chemistry, and 4) either the second semester of organic chemistry or one semester of biochemistry, and predictors of persistence at each milestone. Only 16.5% of students who intended to major in pre-med graduate college with the required coursework for medical schools. Attrition rates are highest initially but drop as students take more relevant courses. Predictors of persistence include academic preparedness before college (e.g., SAT scores, high school GPA) and college performance (e.g., grades in pre-med courses). Students who perform better academically both in high school and in college courses are more likely to remain eligible for medical school.

Keywords: pre-medical studies, attrition, medical education, coursework, college performance

The Process of Attrition in Pre-Medical Studies: A Large-Scale Analysis Across 102 Schools

All students inevitably face the challenge of choosing their vocational path. For many, the process begins with choosing their college major. This is a difficult but extremely important choice with lasting consequences. Some of the most common regrets of Americans involve their educational and career choices (1). The present study investigates a particular case of career planning—the process through which undergraduate students fulfill prerequisite coursework for medical school. It is no secret that a substantial proportion of high school graduates aspire to a career in the medical field. In fact, health professions and related programs were found to be the second most popular field of study among four-year college students in 2018 (2). However, this group of hopeful young adults often change their mind at one point or another throughout their education (3–5). The journey of pursuit in a medical career involves years of strenuous schooling, not to mention the competitive nature of each step along the trajectory. In 2019, applicants to medical schools submitted a median number of 15 applications and only 42.6% of applicants were accepted to any medical program (6,7). Given the lengthy and strenuous journey of pursuit in a medical career involving years of medical school and residency later and the competitive nature of the field, early years of pre-medical studies in undergraduate is no doubt important. The present study investigates the process through which undergraduate students fulfill prerequisite coursework for medical school. Specifically, we examine rates of attrition at different points in the pre-med curriculum and predictors of continual persistence.

A number of studies have examined reasons behind the high rates of attrition in medical education, particularly attrition in medical schools. A systematic review of antecedents for dropouts in medical schools and found substantial effects for lower entry qualifications and poor performance in medical school (8).

While substantial attrition rates have been found in medical schools, past research suggests that attrition begins to occur even earlier. Williams (9) describes the "Pre-Med Syndrome" as the first phase of attrition in the medical education pipeline. However, changing one's career aspirations from medicine to something else in college and dropping out of medical school likely incur very different consequences. Attrition later in the medical education track (e.g., in medical school) is associated with not only much more wasted time spent for students pursuing medicine, but also wasted financial resources associated with high medical training costs (10). Comparatively, attrition in college is associated with much lower costs in both time and resources invested.

Past research has found that upon preparing for medical school application, students are faced with difficult coursework, shattering of unrealistic expectations about what a medical career entails, and harsh admission criteria including high score requirements for the Medical College Assessment Test (MCAT) and college grade point average (GPA). Consistent with this list of challenges, college students report declines in their interest in pre-medical studies (4). Overall, several groups of factors have been linked to attrition in pre-medical studies, namely demographics, academic preparedness, and coursework. We review each of these below.

Certain demographic characteristics have been associated with lower likelihood of persistence in pre-medical studies, specifically being female, and being a member of

underrepresented racial and ethnic minority groups (URM). In a longitudinal study following several cohorts of college freshmen in a single college who indicated interest in pre-medical studies (4), women reported a larger decline in interest in continuing medical education than men, as well as a lower likelihood of applying to medical schools than men. Similar patterns were observed for URM students as compared with non-URM students. However, in another study examining students across six California colleges (11), URM students were found to be almost equal in their likelihood to complete courses required to apply to medical school as non-URM students. On the other hand, demographic characteristics such as having family members who were doctors and higher family income have been found to aid with persistence (4).

Studies have examined whether the gender discrepancy in persistence is coupled with college performance. Fiorentine reported that while males and females with high levels of college performance were equally likely to apply to medical schools, females with low performance were less likely to apply than males with similarly low performance (12). A normative alternatives explanation was proposed such that differences in gender norms provide males with more disincentives to changing their career trajectories when faced with setbacks (13). Consistent with this, Lovecchio and Dundes reported gender to be a moderator for the relationship between performance in organic chemistry courses, with women more likely to alter their career plans as a result of poor performance than men (14). Similarly, pre-medical females construed their own low performance as a sign of poor fit with the medical education track, but not males (15).

Additionally, there has been some suggestion that academic preparedness and performance may influence decisions about persistence in pre-medical education. Among

factors prior to college, high school GPA was found to significantly predict pre-medical student retention (16). However, Barr and colleagues found no association between SAT scores and persisting interest in pre-medical education (4). In a study by Lovecchio and Dundes, 68% of the former pre-med students surveyed pointed to low grades during college as a major concern for their dropping out (14).

When inquired about specific coursework that deterred students from persisting in their interest in medicine in college, frequently mentioned were low grades obtained in difficult pre-medical “gateway” courses, especially the notorious organic chemistry (4,14,17,18).

While this body of research points to demographics, scholastic preparedness, and college performance as predictors of attrition in the pre-medical curriculum, many causes have been derived qualitatively using small-sample interviews and case studies (4,14,15,17–19). Among studies that quantitatively and longitudinally examined predictors of medical education attrition, many used multiple cohorts from a single institution (20). A factor that perhaps can partially account for this lack of large-scale, quantitative research on the undergraduate experience of premed students is that pre-medical studies is not a well-defined major in most post-secondary institutions in the United States. Students who are on the pre-med track often major in biological sciences, physical sciences, health sciences, and some even in humanities and social sciences (21). Therefore, it can be difficult to identify students who are in various majors but are in actuality on the premed track. The present study takes an indirect approach. Rather than attempting to directly identifying the group of pre-med undergraduates, we use all four year of students’ coursework data to distinguish those who do not graduate with the basic pre-requisite

coursework for medical school from those who do. The combination of this coursework criterion and students' self-reported intentions to pursue a pre-med track before let us reasonably estimate the group of pre-med students.

Furthermore, much of the previous work focused on the singular, final status of persisted versus dropped-out. Thus, the approximately four-year process of pre-medical coursework has been glossed over. The 2019 Matriculating Student Questionnaire (MSQ) administered by the Association of American Medical Colleges (AAMC) reported that while a majority of respondents decided that they wanted to study medicine before college, a substantial percentage (34.8%) decided during their four years of college, most of whom (22.1%) decided during the first two years of college (6). There is much to be gained from examining the patterns of attrition throughout the various stages or milestones of achieving a pre-medical degree and completing medical school prerequisite courses.

Our paper focuses on progress through the science prerequisites for medical school among students stating that pre-med is their intent when they take the SAT. Using data collected by the College Board, we have a sample of 15,442 students from 102 post-secondary institutions across the United States for whom we have a complete record of course-taking in college. Based on the required courses for entry into medical school, we are able to examine which students remain medical school-eligible at various milestones: 1) one semester of general chemistry, biology, and physics, 2) two semesters of general chemistry, biology, and physics, 3) one semester of organic chemistry, and 4) either the second semester of organic chemistry or one semester of biochemistry, as well as predictors of fulfillment at each milestone.

We note that our focus here is on whether or not a student stating an initial pre-med intent completes the academic requirements to be eligible to apply to medical school, not on whether the student does or does not apply to medical school. This is a consequence of using a large dataset which contain rich details on individual course-taking provided by 102 colleges and universities. The data are anonymized, prohibiting inquiry into students plans and choices following graduation. This is clearly a limitation of the study, but we view the access to this rare large-scale data base on medical school eligibility as a worthwhile tradeoff.

Method

Sample

Data from students who began their post-secondary education in academic years between 2006 and 2009 were provided by The College Board. Of the 917,459 individuals whose intended major choice information was available, 170,866 individuals had four years of complete coursework data available and attended a school using a standard semester system. Of those, 153,512 students did not indicate any intention in studying pre-medicine at the time of SAT, 1,912 indicated pre-medical studies as their secondary or tertiary major choice, or indicated pre-medical studies as their first choice major but were not certain of their choice, and 15,442 indicated pre-medicine as their first choice major and were very or fairly certain of their choice. This resulted in our primary sample of 15,442 students spanning 102 institutions.

Measures

Demographics. Gender and race/ethnicity information were provided. Of 15,442 students, 9,852 (63.80%) were female and 5,590 (36.20%) were male. Other than 331

(2.01%) individuals whose race/ethnicity information was missing, 8,130 (52.65%) were White, 2,899 (11.39%) were Asian or Pacific Islander, 1,764 (11.42%) were Black or African American, 1,674 (10.84%) were Hispanic, 67 (.43%) were American Indian or Alaska native, and 597 (3.87%) identified as Other race/ethnicity.

Socioeconomic status (SES). Three SES variables were available: father's education, mother's education, and parental income. Parental income was reported on response options that consisted of several income ranges. A dollar value for parental income is calculated by taking the natural logarithm of the midpoint in each income bracket, thus normalizing the distribution. A equally weighted composite of the three variables were calculated by standardizing each variable individually, summing the three, then standardizing the sum again, following the procedure specified by (22).

Intended major choice. Students indicated their intended major choice from a list of 368 different majors at the time they took the SAT.

Degree goal (DEGGOAL). At the time that students took the SAT, they indicated the highest level of education that they intend to pursue. Responses included: 1) specialized training or certificate program; 2) two-year associate of arts or sciences degree; 3) bachelor's degree; 4) master's degree; and 5) doctoral or related degree.

High school GPA (hsGPA). Self-reported GPA was used.

SAT scores. Three SAT scores were provided based on the three subsections: Verbal/Critical Reading (SATV), Writing (SATW), and Math (SATM), with possible scores ranging from 200 to 800. A composite SAT score (SATC) was calculated by summing the three subsection scores.

AP courses. Data were provided on whether students took any AP courses and their grades on the corresponding AP exams, ranging from 1 to 5. The ones relevant to the pre-medicine curriculum were included: biology, chemistry, calculus AB, calculus BC, english language and composition, english literature and composition, physics B, physics C: electricity and magnetism, physics C: mechanics, and statistics.

College coursework. For all college courses that are taken by each student, information was provided about the course name, the year and semester in which the course was taken, the content area in which courses fell, and the grade obtained.

Procedure

Students' eligibility to apply to medical schools (persistence in studying medicine) was operationalized as whether they fulfill the standard coursework required by most medical programs. To determine medical school prerequisites, we tallied definitions used by prior research (11,12,23), admission requirements specified by the Association of American Medical Colleges (AAMC), and course requirements of 105 medical schools across the United States in 2013.

AAMC suggested general prerequisites to be one year of Biology, one year of Physics, and two years of Chemistry that includes Organic Chemistry courses (24). Some medical programs also allowed one biochemistry course to substitute for the second organic chemistry course. Therefore, we operationalized fulfillment of medical school prerequisites to include one year (two semesters) of biology, physics, general chemistry, and either one year of organic chemistry or one semester of organic chemistry with one semester of biochemistry.

The number of courses in each subject was counted to indicate continued eligibility for medical school at a number of milestones during coursework progression. For a course to be included, a numerical grade of 0.7 or higher on a scale of 0 to 4 needed to be achieved, as it is the lowest passing grade.

Further, many students receive college credit for achieving satisfactory grades on AP exams. AP exam scores of 3 or above were accepted by most schools as course credits (25), and were counted toward prerequisite fulfillment.

Analyses

Descriptive statistics were calculated to examine differences between fulfillers of medical school course pre-requisites and non-fulfillers.

Subsequently, logistic regression analyses at several important coursework milestones were performed to examine the effects of demographics, academic preparedness, and college course performance on course fulfillment at each stage.

Results

Rates of Prerequisite Fulfillment

Overall, among the 15,442 students who indicated initial pre-med interests, 2,555 (16.5%) completed the full set of medical school prerequisites, while a small proportion (7.7%) never completed any chemistry, biology, or physics courses. As shown in Fig 1, the process of dropping out is examined at four major milestones, namely

1) whether students took one semester of general chemistry, biology, and physics (39 % did so);
2) among students who fulfilled milestone 1, whether they took a second semester of general chemistry, biology, and physics (64% did so);

3) among students who fulfilled milestone 2, whether they took one semester of organic chemistry (77% did so);

4) among students who fulfilled milestone 3, whether they took a second semester of organic chemistry or a semester of biochemistry, thereby satisfying the entire fulfillment requirement (85% did so).

Fig 1. Pattern of Continuing Fulfillment of Pre-Med Coursework at Various Milestones. Pre-Med Intention = students who indicated intentions of majoring in pre-medicine at the time of taking SAT, Milestone 1 = whether students took a first semester of general chemistry, biology, and physics, Milestone 2 = whether students who completed Milestone1 took a second semester of general chemistry, biology, and physics, Milestone 3 = whether students who completed Milestone2 took a first semester of organic chemistry, Milestone 4 (full fulfillment) = whether students who completed Milestone3 took a second semester of organic chemistry or a semester of biochemistry

To allow comparisons, the number of students who fulfilled prerequisites for medical schools was also tallied for the group of 1,912 students who indicated some intention in studying pre-medicine but were not certain, and the group of 153,512 students who had no intention of studying pre-medicine. 267 (14.0%) fulfilled the full set of medical school prerequisite coursework in the former group and 2,633 (3.9%) fulfilled it in the latter group.

Comparison between Fulfillers and Non-Fulfillers of Medical School Prerequisites

Demographic information of the 2,555 fulfillers and 12,887 non-fulfillers of medical school prerequisites can be found in Table 1. A larger proportion of males (21%) who reported pre-med intentions fulfilled the prerequisites than females (14%).

Table 1 Descriptive Statistics for Gender and Race for Fulfillers and Non-Fulfillers

	Fulfillers			Non-Fulfillers		
Variables	N	Proportion (column)	Proportion (row)	N	Proportion (column)	Proportion (row)
Gender						
Female	1,374	.54	.14	8,478	.66	.86
Male	1,181	.46	.21	4,409	.34	.79
Race						
American Indian	6	.00	.09	61	.00	.91
Asian	675	.27	.23	2,224	.18	.77
Black	165	.07	.09	1,599	.13	.91
Hispanic	210	.08	.13	1,464	.12	.87
White	1,325	.53	.16	6,805	.54	.84
Other	117	.05	.20	480	.04	.80

Note. Fulfillment = two semesters of general chemistry, biology, physics, and organic chemistry or one semester of organic chemistry with one semester of biochemistry, American Indian = American Indian or Alaska Native, Asian = Asian, Asian American, or Pacific Islander, Black = Black or African American, Hispanic = Mexican or Mexican American, Puerto Rican, or Other Hispanic, Latino, or Latin American, Other = Other minorities.

With regards to race/ethnicity, rates of fulfilling prerequisites were 23% for Asians, 20% for Other Minority, 16% for Whites, 13% for Hispanics, and 9% for Blacks.

There were meaningful differences between fulfillers and non-fulfillers on a variety of variables, both prior to college and throughout college (see Table 2). Fulfillers scored higher than non-fulfillers on the SAT-Combined (d = .44), with the largest difference in the Math section (d = .51). Fulfillers also reported higher GPA, both in high school (d = .33), and in all four years of college (average d = .37). Higher socioeconomic status (d = .18) and degree goal (d = .10) were reported by fulfillers than non-fulfillers. In cases where AP grades were available, fulfillers obtained higher AP scores than non-fulfillers, including AP Biology (d = .52), AP Chemistry (d = .38), and AP Physics B and C (average d = .50).

Table 2 Descriptive Statistics for Continuous Variables for Fulfillers and Non-Fulfillers

	Fulfillers			Non-Fulfillers			
Variables	N	Mean	SD	N	Mean	SD	d
SATV	2,516	601.60	84.45	12,748	572.45	87.06	.34
SATM	2,516	636.87	81.81	12,748	591.94	89.32	.51
SATW	2,508	599.53	86.62	12,727	571.09	88.67	.32
SATC	2,508	1838.05	222.62	12,727	1735.54	236.47	.44
HSGPA	2,545	3.96	.32	12,786	3.83	.39	.33
CGPA1	2,552	3.39	.47	12,836	3.07	.60	.54
CGPA2	2,548	3.33	.53	12,706	3.11	.61	.37
CGPA3	2,548	3.37	.56	12,634	3.17	.64	.31
CGPA4	2,542	3.39	.59	12,789	3.21	.70	.27
SES	2,471	.34	.94	12,504	.17	.97	.18
DEGGOAL	2,452	8.87	.74	12,123	8.77	.97	.10
AP Biol	1,167	3.75	1.20	4,204	3.06	1.39	.52
AP Chem	817	3.18	1.29	2,376	2.68	1.35	.38
AP Phys B	303	3.14	1.24	959	2.75	1.29	.31
AP Phys E	70	3.73	1.24	135	2.88	1.42	.62
AP Phys M	146	3.74	1.11	346	3.01	1.33	.57
Biochem N	2,555	.66	.86	12,887	.20	.51	.80
Biol N	2,555	5.99	3.00	12,887	2.56	2.51	1.32
GenChem N	2,555	2.81	1.34	12,887	1.54	1.28	.98
OrgChem N	2,555	2.16	.93	12,887	.44	.80	2.09
Phys N	2,555	2.35	.79	12,887	.72	1.24	1.38
Biochem GR	1,178	3.15	.83	2,020	2.88	.94	.30
Biol GR	2,547	3.16	.68	9,754	2.71	.90	.52
GenChem GR	2,545	3.16	.69	9,382	2.66	.95	.56
OrgChem GR	2,547	2.90	.85	3,746	2.58	.96	.35
Phys GR	2,524	3.20	.75	4,599	2.88	.94	.37

Note. Fulfillment = two semesters of general chemistry, biology, physics, and organic chemistry or one semester of organic chemistry with one semester of biochemistry, d = Cohen's d, SATV = SAT Verbal/Critical Reading, SATM = SAT Mathematics, SATW = SAT Writing, SATC = SAT Composite, HSGPA = self-reported high school GPA, CGPA1 = first-year college GPA, CGPA2 = second-year college GPA, CGPA3 = third-year college GPA, CGPA4 = fourth-year college GPA, SES = socio-economic status, DEGGOAL = degree goal, Biol = Biology, Chem = Chemistry, Phys B = Physics, Phys E = Physics C Electricity & Magnetism, Phys M = Physics C Mechanics, Biochem = Biochemistry, GenChem = General Chemistry, OrgChem = Organic Chemistry, N = number of courses taken in college, GR = grade.

When college performance is broken down specifically by course, fulfillers were found to have taken a greater number of medical school prerequisite courses (average d = 1.31) as well as have performed better in them (average d = .42).

Logistic Regression Models for Predicting Fulfillment Progression

Logistic regression analyses were performed for the four fulfillment milestones. Predictors included gender, ethnicity, the composite SAT score, high school GPA, SES, degree goal, and mean college pre-med course grades when applicable. Continuous variables—SAT, GPA, SES, and course grades—were standardized to aid the comparison between regression coefficients. Variable intercorrelations with each subset of the sample can be found in S1a, b, c, d tables in the supplement. The progression of logistic regression models can be found in Table 3.

Table 3

Summary of Logistic Regression Analysis For Various Milestones Towards Pre-Med Course Fulfillment

	Milestone 1			Milestone 2			Milestone 3			Full Fulfillment (Milestone 4)		
Predictor	*B*	e^B	*SE (B)*	*B*	e^B	*SE (B)*	*B*	e^B	*SE (B)*	*B*	e^B	*SE (B)*
Intercept	-1.00	.37	.18	.12	1.13	.30	.52	1.68	.42	.45	1.56	.55
Gender: Male	.36**	1.44	.04	.19*	1.21	.06	.16	1.17	.09	.21*	1.23	.10
Race: Black	.02	1.02	.06	.12	1.13	.11	-.22	.80	.15	-.31	.73	.19
Race: Asian	.48**	1.62	.05	.04	1.04	.07	.03	1.03	.11	-.04	.96	.12
Race: Hispanic	.04	1.04	.06	.36*	1.44	.11	-.50**	.61	.13	.14	1.15	.19
SATC	.20**	1.22	.02	-.07	.93	.04	.29**	1.33	.05	-.14*	.87	.06
HSGPA	.25**	1.28	.02	-.03	.97	.03	.15*	1.16	.04	.04	1.04	.05
SES	.05*	1.05	.02	-.05	.95	.03	-.06	.94	.05	.02	1.02	.05
DEGGOAL	.04	1.04	.02	.03	1.03	.03	.09	1.09	.05	.09	1.10	.06
Grades				.45**	1.58	.03	.06	1.07	.05	.17*	1.18	.07
OChem										.00	1.00	.07
df	13,048			5,146			3,249			2,496		
pseudo R^2	.07			.06			.06			.02		

Note. Milestone1 = whether students took a first semester of general chemistry, biology, and physics, Milestone2 = whether students who completed Milestone1 took a second semester of general chemistry, biology, and physics, Milestone3 = whether students who completed Milestone2 took a first semester of organic chemistry, Full Fulfillment (Milestone4) = whether students who completed Milestone3 took a second semester of organic chemistry or a semester of biochemistry, Race: Black = Black or African American, Race: Asian = Asian, Asian American, or Pacific Islander, Race: Hispanic: Mexican or Mexican American, Puerto Rican, or Other Hispanic, Latino, or Latin American, SATC = standardized SAT composite, HSGPA = standardized self-reported high school GPA, SES = standardized socio-economic status, DEGGOAL = degree goal, Grades = standardized average grade of all prior courses in general chemistry, biology, and physics, OChem = standardized grade in first-semester organic chemistry, df = residual degrees of freedom, pseudo R^2 = Nagelkerke's R^2 .

*$p < .05$

**$p < .001$

Model 1 examined whether students took first-semester general chemistry, biology, and physics courses with demographic and pre-college predictors. The likelihood of completing this first milestone was 1.44 times greater for males than females, and 1.62 times higher for Asian than White students. Further, students with higher overall scores on SAT (odds ratio (OR) = 1.22), higher high school GPA (OR= 1.28), and higher socioeconomic status (OR = 1.05) were more likely to fulfill these prerequisites.

Model 2 examined whether students who completed a first semester of prerequisites proceeded to complete a second semester of general chemistry, biology, and physics, using not only demographic and pre-college predictors, but also the mean of first-semester general chemistry, biology, and physics grades. Being male (OR = 1.21) and being Hispanic (OR= 1.44) were both associated with higher likelihood of taking a second semester of pre-med courses. Further, one standard deviation increase in average first-semester course grades was related with a 1.58 times greater likelihood to complete these courses.

Subsequently, model 3 used the same set of predictors with course grades computed as the average of grades across both semesters of general chemistry, biology, and physics to determine whether students who have fulfilled milestone 2 took any organic chemistry courses. Hispanic students were only .61 times as likely to take organic chemistry as White students. Both higher SAT scores (OR = 1.33) and higher high school GPA (OR = 1.16) were associated with greater likelihood of completing an organic chemistry course.

Finally, Model 4 used the same previous set of predictors along with the first organic chemistry grade to predict whether students took a second organic chemistry course or a biochemistry course, thereby fulfilling all science coursework prerequisites for medical school, conditional on having completed all required courses thus far. Males were 1.23 times more likely

to complete this last step than females. However, higher SAT scores (OR = .87) were associated with lower likelihood. Lastly, one standard deviation increase in average coursework grades was accompanied by a 1.18 times increase in likelihood of prerequisite completion.

Discussion

The current study examines the pre-medical coursework fulfillment patterns of the group of students who indicated intentions of studying pre-medicine prior to entering college. Only 16.5% of the students graduated with the coursework required by most medical schools. Attrition is highest at early stages and levels off as students commit to the medical education track by taking more of the required courses. Previous studies that found that former pre-med students often mentioned a "distaste for the large pre-med classes" and the highly competitive environment (4), and a change in interest as a result of exposure to other subjects (4,15,18). Thus, while attrition rates in the later college years are comparatively lower and may be attributed to challenging coursework, the initial high attrition may reflect students adjusting their expectations about medicine while discovering interest in non-medical disciplines. This is also consistent with earlier findings that students change their majors often due to interest in and positive perceptions of new major more than negative factors about the old major (26). Given the low acceptance rates into medical schools (7) and the attrition rates in medical schools (3,5), this early change in education track may actually prevent additional personal resources from being wasted in the process of applying to medical programs or institutional resources from being wasted when students drop out of medical programs.

Although a much higher percentage of intended pre-med students completed the full set of medical school prerequisite courses and ended up eligible for medical schools (16.5%) than students with no initial intent (3.9%), the absolute number of the latter group (2,633) was

comparable with that of the former group (2,555). The 2019 MSQ by the AAMC reported that of the medical school matriculants, 55.3% had decided that they wanted to study medicine prior to entering college and 34.8% did so during college (7). When interpreted in light of the present findings, it is evident that a higher percentage of the students who completed medical school prerequisite coursework with initial intent is accepted than without initial intent.

Predictors of Pre-med Persistence

Similar to previous findings on the association between gender and attrition (4,13) and consistent with the normative alternatives approach to explaining the persistence gap (12), being male was linked with a significantly higher likelihood of persisting in a pre-medical education at nearly all stages throughout college. In other words, women's lower likelihood to persist in medicine may be construed as a higher likelihood to accept alternative career choices.

With regards to ethnic and racial identities, while previous investigations suggest that ethnic and racial minority students are less likely to persist (4,11), results of the current study were less consistent. For Asian students, the odds of fulfilling the first semester of coursework were more likely than those of White students, with persistence likelihood decreasing slightly throughout the later milestones but never significantly lower than those of White students. The odds of Hispanic students fulfilling the first year of coursework were higher than those of White students, but the odds of their completing organic chemistry were lower. Lastly, African American students did not differ from White students in their likelihood of persistence at any point.

Further, students who fulfilled all required coursework reported higher SES. Although SES predicted completion of the first semester of required coursework, it did not predict persistence at any of the later milestones. Thus, the advantage of coming from a family with

higher SES found by (4) seems to wear off early on during college. However, its link with persistence in a medical education is likely to strengthen when students decide whether or not to attend medical school, as a $200,000 to $300,000 cost for medical school is no small expense (27).

Consistent with the reputation of a degree in pre-medical studies for being cognitively intensive and challenging, coursework fulfillers entered college with higher scores on all components of the SAT as well as higher high school GPA. Continuing with this advantage, college GPA's of students who eventually fulfilled all required coursework were higher than the GPA's of those who did not for all four years. However, the differences declined over time, with the GPA difference in the fourth year of college being half as large as the GPA difference in the first year of college. This may be an indirect reflection of the high levels of difficulty in pre-medical courses compared with other courses. In terms of college performance, coursework fulfillers both by definition completed a larger number of courses in relevant science subjects and obtained higher grades in them than non-fulfillers.

When examining the predictive validities of academic preparedness measured by variables prior to college (SAT and high school GPA) and college performance (course grades), an interesting pattern is observed. For predicting completion of the first semester of coursework in the absence of any college grades, higher academic preparedness was associated with a greater likelihood of completion. However, the predictive validities of such more distal, pre-college factors were overtaken by that of the more proximal college grades. The exception is whether students complete the first organic chemistry, for which academic preparedness rather than college grades was predictive. This may be due to the notoriously difficult organic chemistry being overwhelmingly identified as culprit in the leaky pipeline (4,14). Students might be extra

cautious in deciding whether they would be able to succeed in the course and resort to information about their academic effectiveness over a longer period of their lives from the past rather than grades from the recent one or two years to make the decision.

Limitations and Future Directions

There are several limitations in the current study. First, because we lacked information about whether students actively pursued pre-medicine throughout college, we focused on completion of coursework required for medical programs. This operationalization is indirect and imperfect. It is possible that students of other science majors completed a similar set of coursework, cases that were considered noise in the current study.

Second, as we were not provided information about whether students who obtained satisfactory scores on AP exams actually used their AP credits toward their college degree, we assumed that any AP exam score of 3 or higher counted as a fulfillment course. There is the possibility that some students may forfeit their AP credits or take the equivalent college course.

Third, since the collection of data used in the present study, a new version of the MCAT that includes a larger social and behavioral science component has been implemented (28). It is reasonable to assume that requirements by medical schools were also revised to contain psychology and sociology coursework. It will be valuable to examine the effects of these changes.

Finally, as our study relied on archival data, we were limited in the extent to which we could explore underlying mechanisms that explain attrition in pre-medical studies. While the regression analyses revealed certain demographic and academic preparedness factors as predictors, no information about the specific reasons behind students' dropping out was

available. However, our study provides a valuable quantitative complement to the prior qualitative body of research that described such reasons.

Conclusion

The present study quantitatively describes the process of attrition as reflected in coursework throughout pre-medical studies in postsecondary institutions. While interventions have been proposed to ameliorate the attrition problem in college, such as improving the undergraduate advising system (4) and reforming the pre-medical curriculum (29,30), our results question the need of any intervention in the very early stages of the medical education track.

Structured Disclosures

Acknowledgements: None.

Funding/Support: None.

Ethical approval: Not applicable.

Disclaimer: None.

Previous presentations: None.

References

1. Roese NJ, Summerville A. What We Regret Most... and Why. Pers Soc Psychol Bull. 2005;31(9):1273–85.

2. Bastrikin A. Number of College Graduates per Year [Internet]. EducationData. 2019 [cited 2020 Apr 5]. Available from: https://educationdata.org/number-of-college-graduates/

3. Arulampalam W, Naylor RA, Smith JP. Dropping out of medical school in the UK: explaining the changes over ten years. Medical Education. 2007;41(4):385–94.

4. Barr DA, Gonzalez ME, Wanat SF. The Leaky Pipeline: Factors Associated With Early Decline in Interest in Premedical Studies Among Underrepresented Minority Undergraduate Students. Academic Medicine. 2008 May;83(5):503.

5. Dyrbye LN, Thomas MR, Power DV, Durning S, Moutier C, Massie FS, et al. Burnout and Serious Thoughts of Dropping Out of Medical School: A Multi-Institutional Study: Academic Medicine. 2010 Jan;85(1):94–102.

6. AAMC. Matriculant Student Questionnaire: 2019 All Schools Summary Report. Association of American Medical Colleges; 2019 p. 26.

7. AAMC. Applicants and Matriculants Data [Internet]. 2018 [cited 2019 Aug 13]. Available from: https://www.aamc.org/data/facts/applicantmatriculant/

8. O'Neill LD, Wallstedt B, Eika B, Hartvigsen J. Factors associated with dropout in medical education: a literature review. Medical Education. 2011;45(5):440–54.

9. Williams D. Craking the Code on the "Hidden Curriculum" in the Medical Education Pipeline and its Contribution to Attrition. 2016;10(19):18.

10. Arulampalam W, Naylor RA, Smith JP. A Hazard Model of the Probability of Medical School Dropout in the United Kingdom. IZA Discussion Papers, No 333, Institute for the Study of Labor (IZA). 2001;44.

11. Alexander C, Chen E, Grumbach K. How Leaky Is the Health Career Pipeline? Minority Student Achievement in College Gateway Courses. Academic Medicine. 2009 Jun;84(6):797.

12. Fiorentine R. Men, Women, and the Premed Persistence Gap: A Normative Alternatives Approach. American Journal of Sociology. 1987 Mar 1;92(5):1118–39.

13. Fiorentine R, Cole S. Why fewer women become physicians: Explaining the premed persistence gap. Sociol Forum. 1992 Sep 1;7(3):469–96.

14. Lovecchio K, Dundes L. Premed Survival: Understanding the Culling Process in Premedical Undergraduate Education. Academic Medicine. 2002 Jul;77(7):719.

15. Grace MK. Parting ways: Sex-based differences in premedical attrition. Social Science & Medicine. 2019 Jun 1;230:222–33.

16. Carter FE. Predictive factors of premedical student retention and degree completion within a private undergraduate university - ProQuest [Internet] [Dissertation]. Mercer University; 2010 [cited 2019 Jun 29]. Available from: https://search.proquest.com/openview/50480e35b487ed7e3d717a34064340fa/1?pq-origsite=gscholar&cbl=18750&diss=y

17. Barr DA, Matsui J, Wanat SF, Gonzalez ME. Chemistry courses as the turning point for premedical students. Adv in Health Sci Educ. 2010 Mar 1;15(1):45–54.

18. Thurmond VB, Cregler LL. Why students drop out of the pipeline to health professions careers: a follow-up of gifted minority high school students. Academic Medicine : Journal of the Association of American Medical Colleges. 1999;74(4):448–451.

19. Gonzalez M, Barr DA, Wanat SF. Attrition From Premedical Studies Among Latinas: Case Studies. Hispanic Journal of Behavioral Sciences. 2010 Nov 1;32(4):571–85.

20. Yates J, James D. Risk factors for poor performance on the undergraduate medical course: cohort study at Nottingham University. Medical Education. 2007;41(1):65–73.

21. AAMC. How to choose the best premed major [Internet]. Association of American Medical Colleges. 1995 [cited 2020 Apr 5]. Available from: https://students-residents.aamc.org/applying-medical-school/article/how-choose-best-premed-major/

22. Sackett PR, Kuncel NR, Arneson JJ, Cooper SR, Waters SD. Does socioeconomic status explain the relationship between admissions tests and post-secondary academic performance? Psychological bulletin. 2009;135(1):1.

23. Liboff AR, Chopp M. Should the premed requirements in physics be changed? American Journal of Physics. 1979 Apr 1;47(4):331–6.

24. Association of American Medical Colleges. Admission Requirements [Internet]. Association of American Medical Colleges. 2019 [cited 2019 Jul 4]. Available from: https://students-residents.aamc.org/applying-medical-school/applying-medical-school-process/medical-school-admission-requirements/admission-requirements/

25. College Board. AP Credit Policy Search [Internet]. College Board. 2019 [cited 2019 Jun 29]. Available from: https://apstudents.collegeboard.org/getting-credit-placement/search-policies

26. Malgwi CA, Howe MA, Burnaby PA. Influences on Students' Choice of College Major. Journal of Education for Business. 2005 May;80(5):275–82.

27. Association of American Medical Colleges. Financial Aid [Internet]. Association of American Medical Colleges. 2019 [cited 2019 Jun 29]. Available from: https://students-residents.aamc.org/financial-aid/

28. Frazer NB, Twohig MP. The New Social and Behavioral MCAT Requirements: Inspired Innovation, Missed Opportunity, or Both? Peer Review. 2012;14(4):14–17.

29. Dienstag JL. Relevance and Rigor in Premedical Education. The New England Journal of Medicine. 2008;359:221–224.

30. Emanuel EJ. Changing premed requirements and the medical curriculum. JAMA. 2006;296(9):1128–1131.

Manuscript

Click here to access/download;Manuscript;Revised Manuscript without Track Changes_201015.docx

The process of attrition in pre-medical studies: A large-scale analysis across 102 schools

Short title: Attrition in pre-medical studies

Charlene Zhang (B.S.)—Corresponding Author
University of Minnesota
75 E River Rd #N496, Minneapolis, MN 55455
zhan5449@umn.edu
Tel: 612-839-2310

Nathan R. Kuncel (Ph.D.)
University of Minnesota
75 E River Rd #N218, Minneapolis, MN 55455
kunce001@umn.edu
Tel: 612-624-1651

Paul R. Sackett (Ph.D.)
University of Minnesota
75 E River Rd #N475, Minneapolis, MN 55455
psackett@umn.edu
Tel: 612-624-9842

Abstract

The important but difficult choice of vocational trajectory often takes place in college, beginning with majoring in a subject and taking relevant coursework. Of all possible disciplines, pre-medical studies are often not a formally defined major but pursued by a substantial proportion of the college population. Understanding students' experiences with pre-med coursework is valuable and understudied, as most research on medical education focuses on the later medical school and residency. We examined the pattern and predictors of attrition at various milestones along the pre-med coursework track during college. Using a College Board dataset, we analyzed a sample of 15,442 students spanning 102 institutions who began their post-secondary education in years between 2006 and 2009. We examined whether students fulfilled the required coursework to remain eligible for medical schools at several milestones: 1) one semester of general chemistry, biology, physics, 2) two semesters of general chemistry, biology, physics, 3) one semester of organic chemistry, and 4) either the second semester of organic chemistry or one semester of biochemistry, and predictors of persistence at each milestone. Only 16.5% of students who intended to major in pre-med graduate college with the required coursework for medical schools. Attrition rates are highest initially but drop as students take more advanced courses. Predictors of persistence include academic preparedness before college (e.g., SAT scores, high school GPA) and college performance (e.g., grades in pre-med courses). Students who perform better academically both in high school and in college courses are more likely to remain eligible for medical school.

Keywords: pre-medical studies, attrition, medical education, coursework, college performance

The Process of Attrition in Pre-Medical Studies: A Large-Scale Analysis Across 102 Schools

All students inevitably face the challenge of choosing their vocational path. For many, the process begins with choosing their college major. This is a difficult but extremely important choice with lasting consequences. Some of the most common regrets of Americans involve their educational and career choices (1). The present study investigates a particular case of career planning—the process through which undergraduate students fulfill prerequisite coursework for medical school. It is no secret that a substantial proportion of high school graduates aspire to a career in the medical field. In fact, health professions and related programs were found to be the second most popular field of study among four-year college students in 2018 (2). However, this group of hopeful young adults often change their mind at one point or another throughout their education (3,4). The journey of pursuit in a medical career involves years of strenuous schooling, not to mention the competitive nature of each step along the trajectory. In 2019, applicants to medical schools submitted a median number of 15 applications and only 42.6% of applicants were accepted to any medical program (5,6). Given the lengthy and strenuous journey of pursuit in a medical career involving years of medical school and residency later and the competitive nature of the field, early years of pre-medical studies in undergraduate is no doubt important. The present study investigates the process through which undergraduate students fulfill prerequisite coursework for medical school. Specifically, we examine rates of attrition at different points in the pre-med curriculum and predictors of continual persistence.

Williams (7) describes the “Pre-Med Syndrome” as the first phase of attrition in the medical education pipeline. Past research has found that upon preparing for medical school application, students are faced with difficult coursework, shattering of unrealistic expectations

about what a medical career entails, and harsh admission criteria including high score requirements for the Medical College Assessment Test (MCAT) and college grade point average (GPA). Consistent with this list of challenges, college students report declines in their interest in pre-medical studies (3). Overall, several groups of factors have been linked to attrition in pre-medical studies, namely demographics, academic preparedness, and coursework. We review each of these below.

Certain demographic characteristics have been associated with lower likelihood of persistence in pre-medical studies, specifically being female, and being a member of underrepresented racial and ethnic minority groups (URM). In a longitudinal study following several cohorts of college freshmen in a single college who indicated interest in pre-medical studies (3), women reported a larger decline in interest in continuing medical education than men, as well as a lower likelihood of applying to medical schools than men. Similar patterns were observed for URM students as compared with non-URM students. However, in another study examining students across six California colleges (8), URM students were found to be almost equal in their likelihood to complete courses required to apply to medical school as non-URM students. On the other hand, demographic characteristics such as having family members who were doctors and higher family income have been found to aid with persistence (3).

Studies have examined whether the gender discrepancy in persistence is coupled with college performance. Fiorentine reported that while males and females with high levels of college performance were equally likely to apply to medical schools, females with low performance were less likely to apply than males with similarly low performance (9). A normative alternatives explanation was proposed such that differences in gender norms provide males with more disincentives to changing their career trajectories when faced with setbacks

(10). Consistent with this, Lovecchio and Dundes reported gender to be a moderator for the relationship between performance in organic chemistry courses, with women more likely to alter their career plans as a result of poor performance than men (11). Similarly, pre-medical females construed their own low performance as a sign of poor fit with the medical education track, but not males (12).

The issue of underrepresentation of students from URM groups in medicine and even the broader science, technology, engineering, and mathematics (STEM) field is systemic, beginning with being less prepared in pre-college and college courses (13). Less access to and participation in AP science courses, lack of support and guidance from family and faculty mentors, and financial challenges can all affect whether students from URM persist (14–16).

Additionally, there has been some suggestion that academic preparedness and performance may influence decisions about persistence in pre-medical education. Among factors prior to college, high school GPA was found to significantly predict pre-medical student retention (17). However, Barr and colleagues found no association between SAT scores and persisting interest in pre-medical education (3). In a study by Lovecchio and Dundes, 68% of the former pre-med students surveyed pointed to low grades during college as a major concern for their dropping out (11).

When inquired about specific coursework that deterred students from persisting in their interest in medicine in college, frequently mentioned were low grades obtained in difficult pre-medical “gateway” courses, especially the notorious organic chemistry (3,11,18,19). Further, the discouraging effects of such chemistry course have been found to be especially pronounced for students from URM groups and women (3,18,20).

While this body of research points to demographics, scholastic preparedness, and college performance as predictors of attrition in the pre-medical curriculum, many causes have been derived qualitatively using small-sample interviews and case studies (3,11,12,18,19,21). Among studies that quantitatively and longitudinally examined predictors of medical education attrition, many used multiple cohorts from a single institution (22). A factor that perhaps can partially account for this lack of large-scale, quantitative research on the undergraduate experience of premed students is that pre-medical studies is not a well-defined major in most post-secondary institutions in the United States. Students who are on the pre-med track often major in biological sciences, physical sciences, health sciences, and some even in humanities and social sciences (23). Therefore, it can be difficult to identify students who are in various majors but are in actuality on the premed track. The present study takes an indirect approach. Rather than attempting to directly identifying the group of pre-med undergraduates, we use all four year of students' coursework data to distinguish those who do not graduate with the basic pre-requisite coursework for medical school from those who do. The combination of this coursework criterion and students' self-reported intentions to pursue a pre-med track before let us reasonably estimate the group of pre-med students.

Furthermore, much of the previous work focused on the singular, final status of persisted versus dropped-out. Thus, the approximately four-year process of pre-medical coursework has been glossed over. The 2019 Matriculating Student Questionnaire (MSQ) administered by the Association of American Medical Colleges (AAMC) reported that while a majority of respondents decided that they wanted to study medicine before college, a substantial percentage (34.8%) decided during their four years of college, most of whom (22.1%) decided during the first two years of college (5). There is much to be gained from examining the patterns of attrition

throughout the various stages or milestones of achieving a pre-medical degree and completing medical school prerequisite courses.

Our paper focuses on progress through the science prerequisites for medical school among students stating that pre-med is their intent when they take the SAT. Using data collected by the College Board, we have a sample of 15,442 students from 102 post-secondary institutions across the United States for whom we have a complete record of course-taking in college. Based on the required courses for entry into medical school, we are able to examine which students remain medical school-eligible at various milestones: 1) one semester of general chemistry, biology, and physics, 2) two semesters of general chemistry, biology, and physics, 3) one semester of organic chemistry, and 4) either the second semester of organic chemistry or one semester of biochemistry, as well as predictors of fulfillment at each milestone.

We note that our focus here is on whether or not a student stating an initial pre-med intent completes the academic requirements to be eligible to apply to medical school, not on whether the student does or does not apply to medical school. This is a consequence of using a large dataset which contain rich details on individual course-taking provided by 102 colleges and universities. The data are anonymized, prohibiting inquiry into students plans and choices following graduation. This is clearly a limitation of the study, but we view the access to this rare large-scale data base on medical school eligibility as a worthwhile tradeoff.

Method

Sample

Data from students who began their post-secondary education in academic years between 2006 and 2009 were provided by The College Board. Of the 917,459 individuals whose intended major choice information was available, 170,866 individuals had four years of complete

coursework data available and attended a school using a standard semester system. Of those, 153,512 students did not indicate any intention in studying pre-medicine at the time of SAT, 1,912 indicated pre-medical studies as their secondary or tertiary major choice, or indicated pre-medical studies as their first choice major but were not certain of their choice, and 15,442 indicated pre-medicine as their first choice major and were very or fairly certain of their choice. This resulted in our primary sample of 15,442 students spanning 102 institutions.

Measures

Demographics. Gender and race/ethnicity information were provided. Of 15,442 students, 9,852 (63.80%) were female and 5,590 (36.20%) were male. Other than 331 (2.01%) individuals whose race/ethnicity information was missing, 8,130 (52.65%) were White, 2,899 (11.39%) were Asian or Pacific Islander, 1,764 (11.42%) were Black or African American, 1,674 (10.84%) were Hispanic, 67 (.43%) were American Indian or Alaska native, and 597 (3.87%) identified as Other race/ethnicity.

Socioeconomic status (SES). Three SES variables were available: father’s education, mother’s education, and parental income. Parental income was reported on response options that consisted of several income ranges. A dollar value for parental income is calculated by taking the natural logarithm of the midpoint in each income bracket, thus normalizing the distribution. A equally weighted composite of the three variables were calculated by standardizing each variable individually, summing the three, then standardizing the sum again, following the procedure specified by (24).

Intended major choice. Students indicated their intended major choice from a list of 368 different majors at the time they took the SAT.

High school GPA (hsGPA). Self-reported GPA was used.

SAT scores. Three SAT scores were provided based on the three subsections: Verbal/Critical Reading (SATV), Writing (SATW), and Math (SATM), with possible scores ranging from 200 to 800. A composite SAT score (SATC) was calculated by summing the three subsection scores.

AP courses. Data were provided on whether students took any AP courses and their grades on the corresponding AP exams, ranging from 1 to 5. The ones relevant to the pre-medicine curriculum were included: biology, chemistry, calculus AB, calculus BC, english language and composition, english literature and composition, physics B, physics C: electricity and magnetism, physics C: mechanics, and statistics.

College coursework. For all college courses that are taken by each student, information was provided about the course name, the year and semester in which the course was taken, the content area in which courses fell, and the grade obtained.

Procedure

Students' eligibility to apply to medical schools (persistence in studying medicine) was operationalized as whether they fulfill the standard coursework required by most medical programs. To determine medical school prerequisites, we tallied definitions used by prior research (8,9,25), admission requirements specified by the Association of American Medical Colleges (AAMC), and course requirements of 105 medical schools across the United States in 2013.

AAMC suggested general prerequisites to be one year of Biology, one year of Physics, and two years of Chemistry that includes Organic Chemistry courses (26). Some medical programs also allowed one biochemistry course to substitute for the second organic chemistry course. Therefore, we operationalized fulfillment of medical school prerequisites to include one

year (two semesters) of biology, physics, general chemistry, and either one year of organic chemistry or one semester of organic chemistry with one semester of biochemistry.

The number of courses in each subject was counted to indicate continued eligibility for medical school at a number of milestones during coursework progression. For a course to be included, a numerical grade of 0.7 or higher on a scale of 0 to 4 needed to be achieved, as it is the lowest passing grade.

Further, many students receive college credit for achieving satisfactory grades on AP exams. AP exam scores of 3 or above were accepted by most schools as course credits (27), and were counted toward prerequisite fulfillment.

Analyses

Descriptive statistics were calculated to examine differences between fulfillers of medical school course pre-requisites and non-fulfillers.

Subsequently, logistic regression analyses at several important coursework milestones were performed to examine the effects of demographics, academic preparedness, and college course performance on course fulfillment at each stage.

Results

Rates of Prerequisite Fulfillment

Overall, among the 15,442 students who indicated initial pre-med interests, 2,555 (16.5%) completed the full set of medical school prerequisites, while a small proportion (7.7%) never completed any chemistry, biology, or physics courses. As shown in Fig 1, the process of dropping out is examined at four major milestones, namely

1) whether students passed one semester of general chemistry, biology, and physics (39 % did so);

2) among students who fulfilled milestone 1, whether they passed a second semester of general chemistry, biology, and physics (64% did so);

3) among students who fulfilled milestone 2, whether they passed one semester of organic chemistry (77% did so);

4) among students who fulfilled milestone 3, whether they passed a second semester of organic chemistry or a semester of biochemistry, thereby satisfying the entire fulfillment requirement (85% did so).

Fig 1. Pattern of Continuing Fulfillment of Pre-Med Coursework at Various Milestones. Pre-Med Intention = students who indicated intentions of majoring in pre-medicine at the time of taking SAT, Milestone 1 = whether students took a first semester of general chemistry, biology, and physics, Milestone 2 = whether students who completed Milestone1 took a second semester of general chemistry, biology, and physics, Milestone 3 = whether students who completed Milestone2 took a first semester of organic chemistry, Milestone 4 (full fulfillment) = whether students who completed Milestone3 took a second semester of organic chemistry or a semester of biochemistry

Proportions of fulfillers at each milestone were also calculated for each of the 102 institutions individually. Across all institutions, an average of 34% of students fulfilled milestone 1 (SD = 19%), 23% fulfilled milestone 2 (SD = 16%), 19% fulfilled milestone 3 (SD = 16%), and 17% fulfilled milestone 4 (SD = 16%).

To allow comparisons, the number of students who fulfilled prerequisites for medical schools was also tallied for the group of 1,912 students who indicated some intention in studying pre-medicine but were not certain, and the group of 153,512 students who had no intention of studying pre-medicine. 267 (14.0%) fulfilled the full set of medical school prerequisite coursework in the former group and 2,633 (3.9%) fulfilled it in the latter group.

Comparison between Fulfillers and Non-Fulfillers of Medical School Prerequisites

Demographic information of the 2,555 fulfillers and 12,887 non-fulfillers of medical school prerequisites can be found in Table 1. A larger proportion of males (21%) who reported pre-med intentions fulfilled the prerequisites than females (14%).

Table 1. Sample Size and Percentage of Prerequisite Fulfillers at Each Milestone by Gender and Race

		Percentage Within Group that Fulfilled Milestone			
Group	n	M1	M2	M3	M4
Overall	15,442	39.35%	25.06%	19.36%	16.55%
Gender					
Male	5,590	45.65%	30.41%	23.94%	21.13%
Female	9,852	35.78%	22.02%	16.77%	13.95%
Race					
White	8,130	38.18%	24.24%	19.11%	16.30%
Asian	2,899	50.85%	32.63%	26.11%	23.28%
Black	1,764	30.05%	17.74%	12.24%	9.35%
Hispanic	1,674	33.63%	22.70%	15.11%	12.54%
American Indian	67	22.39%	16.42%	11.94%	8.96%
Other	597	44.39%	28.48%	22.95%	19.60%
Race x Gender					
White Male	3,080	43.67%	29.16%	23.54%	20.65%
White Female	5,050	34.83%	21.25%	16.42%	13.64%
Asian Male	1,205	53.69%	35.02%	27.88%	26.14%
Asian Female	1,694	48.82%	30.93%	24.85%	21.25%
Black Male	353	39.09%	24.93%	17.00%	12.75%
Black Female	1,411	27.78%	15.95%	11.06%	8.50%
Hispanic Male	586	41.30%	30.72%	21.67%	18.09%
Hispanic Female	1,088	29.50%	18.38%	11.58%	9.56%
American Indian Male	24	16.67%	12.50%	8.33%	8.33%
American Indian Female	43	25.58%	18.60%	13.95%	9.30%
Other Male	213	53.99%	32.86%	25.35%	22.54%
Other Female	384	39.06%	26.04%	21.61%	17.97%

Notes. M1 = Milestone 1, including a first semester of general chemistry, biology, and physics, M2 = Milestone 2, including two semesters of general chemistry, biology, and physics, M3 = Milestone 3, including Milestone2 as well as a first semester of organic chemistry, M4 = Milestone 4 (full fulfillment), including Milestone 3 as well as a second semester of organic chemistry or a semester of biochemistry. American Indian = American Indian or Alaska Native, Asian = Asian, Asian American, or Pacific Islander, Black = Black or African

American, Hispanic = Mexican or Mexican American, Puerto Rican, or Other Hispanic, Latino, or Latin American, Other = Other minorities.

With regards to race/ethnicity, rates of fulfilling prerequisites among those who had intention of pursuing pre-medical studies were the highest for Asians (23%), followed by the Other Minority group (20%), then Whites (16%) and Hispanics (13%), and the lowest for Blacks (9%).

There were meaningful differences between fulfillers and non-fulfillers on a variety of variables, both prior to college and throughout college (see Table 2). Fulfillers scored higher than non-fulfillers on the SAT-Combined (*d* = .44), with the largest difference in the Math section (*d* = .51). Fulfillers also reported higher GPA, both in high school (*d* = .33), and in all four years of college (average *d* = .37). Higher socioeconomic status (*d* = .18) was reported by fulfillers than non-fulfillers. In cases where AP grades were available, fulfillers obtained higher AP scores than non-fulfillers, including AP Biology (*d* = .52), AP Chemistry (*d* = .38), and AP Physics B and C (average *d* = .50).

Table 2 Descriptive Statistics for Continuous Variables for Fulfillers and Non-Fulfillers

	Fulfillers			Non-Fulfillers			
Variables	N	Mean	SD	N	Mean	SD	d
SAT-Verbal	2,516	601.60	84.45	12,748	572.45	87.06	.34
SAT-Math	2,516	636.87	81.81	12,748	591.94	89.32	.51
SAT-Writing	2,508	599.53	86.62	12,727	571.09	88.67	.32
SAT-Composite	2,508	1838.05	222.62	12,727	1735.54	236.47	.44
High School GPA	2,545	3.96	.32	12,786	3.83	.39	.33
SES	2,471	.34	.94	12,504	.17	.97	.18
AP grades							
AP Biol	1,167	3.75	1.20	4,204	3.06	1.39	.52
AP Chem	817	3.18	1.29	2,376	2.68	1.35	.38
AP Phys B	303	3.14	1.24	959	2.75	1.29	.31

AP Phys E	70	3.73	1.24	135	2.88	1.42	.62
AP Phys M	146	3.74	1.11	346	3.01	1.33	.57
Number of courses taken							
Biochem N	2,555	.66	.86	12,887	.20	.51	.32
Biol N	2,555	5.99	3.00	12,887	2.56	2.51	.65
GenChem N	2,555	2.81	1.34	12,887	1.54	1.28	.53
OrgChem N	2,555	2.16	.93	12,887	.44	.80	.83
Phys N	2,555	2.35	.79	12,887	.72	1.24	.77
College grades							
Biochem GR	1,178	3.15	.83	2,020	2.88	.94	.30
Biol GR	2,547	3.16	.68	9,754	2.71	.90	.52
GenChem GR	2,545	3.16	.69	9,382	2.66	.95	.56
OrgChem GR	2,547	2.90	.85	3,746	2.58	.96	.35
Phys GR	2,524	3.20	.75	4,599	2.88	.94	.37

Note. Fulfillment = two semesters of general chemistry, biology, physics, and organic chemistry or one semester of organic chemistry with one semester of biochemistry, d = Cliff's δ for number of courses and Cohen's d for all other variables, SES = socio-economic status, Biol = Biology, Chem = Chemistry, Phys B = Physics, Phys E = Physics C Electricity & Magnetism, Phys M = Physics C Mechanics, Biochem = Biochemistry, GenChem = General Chemistry, OrgChem = Organic Chemistry.

When college performance is broken down specifically by course, fulfillers were found to have taken a greater number of medical school prerequisite courses (average *d* = 1.31) as coursework definitionally distinguishes fulfillers from non-fulfillers. They were also found to have performed better in these courses (average *d* = .42).

Logistic Regression Models for Predicting Fulfillment Progression

Logistic regression analyses were performed for the four fulfillment milestones. Predictors included gender, ethnicity, the composite SAT score, high school GPA, SES, and mean college pre-med course grades when applicable. Continuous variables—SAT, GPA, SES, and course grades—were standardized to aid the comparison between regression coefficients. Variable intercorrelations with each subset of the sample can be found in S1, 2, 3, and 4 tables in the supplement. The progression of logistic regression models can be found in Table 3. The same

models with individual prior course grades as predictors were also tested and yielded similar results.

Table 3 reports the logistic regression coefficients as well as the effect sizes in odds ratios (OR). To aid the interpretation of these results, we also computed predicted likelihoods based on the regression weights. To compute fulfillment likelihoods of various demographic groups, we plugged in average values of all other predictors. To compute the continuous variables' effects on fulfillment likelihoods, we plugged in the reference groups (i.e., females for gender, White for race), average values of all other predictors, and the average and 1 standard deviation (SD) above average values of the predictor of interest.

Table 3

Summary of Logistic Regression Analysis For Various Milestones Towards Pre-Med Course Fulfillment

	Milestone 1			Milestone 2			Milestone 3			Full Fulfillment (Milestone 4)		
Predictor	*B*	e^B	*SE (B)*	*B*	e^B	*SE (B)*	*B*	e^B	*SE (B)*	*B*	e^B	*SE (B)*
Intercept	-.70**	.50	.03	.42**	1.52	.05	1.25**	3.48	.07	1.28**	3.59	.08
Gender: Male	.36**	1.44	.04	.19**	1.21	.06	.14	1.15	.08	.17	1.18	.10
Race: Black	.04	1.05	.06	.14	1.15	.11	-.15	.86	.15	-.30	.74	.18
Race: Asian	.50**	1.65	.05	.07	1.07	.07	.02	1.02	.10	-.07	.93	.11
Race: Hispanic	.04	1.04	.06	.37**	1.44	.11	-.44**	.64	.13	.16	1.17	.18
SATC	.21**	1.23	.02	-.05	.95	.04	.28**	1.33	.05	-.13*	.88	.06
HSGPA	.24**	1.28	.02	-.03	.97	.03	.15**	1.17	.04	.03	1.03	.05
SES	.04*	1.04	.02	-.05	.95	.03	-.05	.95	.04	.01	1.01	.05
Grades				.44**	1.56	.03	.07	1.07	.05	.19*	1.21	.07
OChem										-.01	.99	.07
df	13,786			5,404			3,419			2,610		
pseudo R^2	.07			.06			.05			.02		
AIC	17,768			6,903			3,603			2,704		

Note. Reference group for Gender was Female. Reference group for Race was White. Milestone1 = whether students took a first semester of general chemistry, biology, and physics, Milestone2 = whether students who completed Milestone1 took a second semester of general chemistry, biology, and physics, Milestone3 = whether students who completed Milestone2 took a first semester of organic chemistry, Full Fulfillment (Milestone4) = whether students who completed Milestone3 took a second semester of organic chemistry or a semester of biochemistry, Race: Black = Black or African American, Race: Asian = Asian, Asian American, or Pacific Islander, Race: Hispanic: Mexican or Mexican American, Puerto Rican, or Other Hispanic, Latino, or Latin American, SATC = standardized SAT composite, HSGPA = standardized self-reported high school GPA, SES = standardized socio-economic status, Grades = standardized average grade of all prior courses in general chemistry, biology, and physics, OChem = standardized grade in first-semester organic chemistry, df = residual degrees of freedom, pseudo R^2 = Nagelkerke's R^2 .

*$p < .05$

**$p < .001$

Model 1 examined whether students took first-semester general chemistry, biology, and physics courses with demographic and pre-college predictors (milestone 1). The predicted likelihood of males fulfilling milestone 1 was 8.50% higher than that of females (odds ratio (OR) = 1.44), and the likelihood of Asian students completing the milestone was 11.97% higher than that of White students (OR = 1.65), net of all other predictors. Further, controlling for all other predictors, the predicted likelihood of fulfilling milestone 1 increased by 4.82% with every 1 SD increase in SAT score (OR = 1.23), 5.63% with every 1SD increase in high school GPA (OR = 1.28), and .98% with every 1SD increase in SES (OR = 1.04).

Model 2 examined whether students who completed a first semester of prerequisites proceeded to complete a second semester of general chemistry, biology, and physics (milestone 2), using not only demographic and pre-college predictors, but also the mean of first-semester general chemistry, biology, and physics grades. Among students who had fulfilled milestone 1, the predicted likelihood of males fulfilling milestone 2 was 4.48% higher than that of females (OR = 1.21), and the likelihood of Hispanic students was 8.34% higher than that of White females (OR = 1.44) when holding all other predictors constant. Further, 1SD increase in average first-semester course grades improved the likelihood of students fulfilling milestone 2 by 10.04% net of all other predictors (OR = 1.56).

Subsequently, model 3 used the same set of predictors with course grades computed as the average of grades across both semesters of general chemistry, biology, and physics to determine whether students who have fulfilled milestone 2 took any organic chemistry courses (milestone 3). Among students who had completed milestone 2, the likelihood of Hispanic students taking and passing an organic chemistry course was 8.58% lower than that of White females when holding all other predictors constant at average (OR = .64). The likelihood of

fulfilling milestone 3 also increased by 4.51% with every 1SD increase in SAT score (OR = 1.33) and by 2.54% with every 1SD increase in high school GPA (OR = 1.17), net of all other variables.

Finally, Model 4 used the same previous set of predictors along with the first organic chemistry grade to predict whether students took a second organic chemistry course or a biochemistry course, thereby fulfilling all science coursework prerequisites for medical school, conditional on having completed all required courses thus far (milestone 4). There were no statistically significant difference in fulfillment likelihood between gender and racial majority and minority groups. Controlling for all other predictors, 1SD increase SAT scores decreased predicted likelihood of prerequisite completion by 2.24% (OR = .88), and 1SD increase in average coursework grades increased likelihood by 3.09% (OR = 1.21).

To explore the potential demographic variables' interactions in predicting coursework fulfillment likelihoods, the same four models were also tested including the interaction terms between gender and race dummy variables. The only statistically significant and meaningful interaction was between being male and being Asian for fulfilling coursework milestone 2 (OR = .71, $p < .001$) and milestone 3 (OR = .69, $p < .001$). In other words, holding all other predictors constant at their average values, while the predicted likelihood of White males fulfilling milestone 2 exceeds that of White females by 8.67%, the difference in likelihood between Asian males and females is only 2.14%. Similarly, the difference between White males' and females' predicted likelihood for completing milestone 3 is 7.81%, while that between Asian males and females is 1.53%.

Additional logistic regression models with various interactions terms between demographic dummy variable (i.e., gender and race) and the continuous predictors (i.e., SAT,

high school GPA, SES, and grades) were also tested individually. For predicting milestones 1, 2, and 3, there was a significant interaction between gender and SAT score such that the difference in fulfillment likelihood between males and females (in favor of males) was reduced as SAT score increased (OR = .88, .84, and .88, respectively). A similar effect was found in the White–Asian comparison for milestones 1 and 2, such that the higher fulfillment likelihood of Asian students than White students was reduced with higher SAT scores (OR = .83 and .84, respectively). In addition, for predicting fulfillment of milestone 3, the higher likelihood of Asian than White students was reduced as average college course grade increased (OR = .88). Lastly, the higher likelihood of Asian students fulfilling milestone 4 than White students was enhanced with higher high school GPA (OR = 1.31).

Discussion

The current study examines the pre-medical coursework fulfillment patterns of the group of students who indicated intentions of studying pre-medicine prior to entering college. Only 16.5% of the students graduated with the coursework required by most medical schools. Attrition is highest at early stages and levels off as students commit to the medical education track by taking more of the required courses. Previous studies that found that former pre-med students often mentioned a “distaste for the large pre-med classes” and the highly competitive environment (3), and a change in interest as a result of exposure to other subjects (3,12,19). Thus, while attrition rates in the later college years are comparatively lower and may be attributed to challenging coursework, the initial high attrition may reflect students adjusting their expectations about medicine while discovering interest in non-medical disciplines. This is also consistent with earlier findings that students change their majors often due to interest in and positive perceptions of new major more than negative factors about the old major (28). Given the

low acceptance rates into medical schools (6) and the attrition rates in medical schools (4,29), this early change in education track may actually prevent additional personal resources from being wasted in the process of applying to medical programs or institutional resources from being wasted when students drop out of medical programs.

Although a much higher percentage of intended pre-med students completed the full set of medical school prerequisite courses and ended up eligible for medical schools (16.5%) than students with no initial intent (3.9%), the absolute number of the latter group (2,633) was comparable with that of the former group (2,555). The 2019 MSQ by the AAMC reported that of the medical school matriculants, 55.3% had decided that they wanted to study medicine prior to entering college and 34.8% did so during college (6). When interpreted in light of the present findings, it is evident that a higher percentage of the students who completed medical school prerequisite coursework with initial intent is accepted than without initial intent.

Predictors of Pre-med Persistence

Similar to previous findings on the association between gender and attrition (3,10) and consistent with the normative alternatives approach to explaining the persistence gap (9), being male was linked with a significantly higher likelihood of persisting in a pre-medical education at nearly all stages throughout college. In other words, women's lower likelihood to persist in medicine may be construed as a higher likelihood to accept alternative career choices.

With regards to ethnic and racial identities, while previous investigations suggest that ethnic and racial minority students are less likely to persist (3,8), results of the current study were less consistent. For Asian students, the odds of fulfilling the first semester of coursework were more likely than those of White students, with persistence likelihood decreasing slightly throughout the later milestones but never significantly lower than those of White students. The

odds of Hispanic students fulfilling the first year of coursework were higher than those of White students, but the odds of their completing organic chemistry were lower. Lastly, African American students did not differ from White students in their likelihood of persistence at any point after controlling for socio-economic status, SAT, and grades in high school and college.

Further, students who fulfilled all required coursework reported higher SES. Although SES predicted completion of the first semester of required coursework, it did not predict persistence at any of the later milestones. Thus, the advantage of coming from a family with higher SES found by (3) seems to wear off early on during college. However, its link with persistence in a medical education is likely to strengthen when students decide whether or not to attend medical school, as a $200,000 to $300,000 cost for medical school is no small expense (30).

Consistent with the reputation of a degree in pre-medical studies for being cognitively intensive and challenging, coursework fulfillers entered college with higher scores on all components of the SAT as well as higher high school GPA. Continuing with this advantage, college GPA's of students who eventually fulfilled all required coursework were higher than the GPA's of those who did not for all four years. However, the differences declined over time, with the GPA difference in the fourth year of college being half as large as the GPA difference in the first year of college. This may be an indirect reflection of the high levels of difficulty in pre-medical courses compared with other courses. In terms of college performance, coursework fulfillers both by definition completed a larger number of courses in relevant science subjects and obtained higher grades in them than non-fulfillers.

When examining the predictive validities of academic preparedness measured by variables prior to college (SAT and high school GPA) and college performance (course grades),

an interesting pattern is observed. For predicting completion of the first semester of coursework in the absence of any college grades, higher academic preparedness was associated with a greater likelihood of completion. However, the predictive validities of such more distal, pre-college factors were overtaken by that of the more proximal college grades. The exception is whether students complete the first organic chemistry, for which academic preparedness rather than college grades was predictive. This may be due to the notoriously difficult organic chemistry being overwhelmingly identified as culprit in the leaky pipeline (3,11). Students might be extra cautious in deciding whether they would be able to succeed in the course and resort to information about their academic effectiveness over a longer period of their lives from the past rather than grades from the recent one or two years to make the decision. In a recent study examining persistence in undergraduate STEM courses, it was found that grades in the first general chemistry course was related with subsequent persistence in STEM more strongly among underrepresented individuals than among well-represented students (20). Our examination of such interactions between demographic group and other predictors (e.g., academic preparedness before college, grades in college) yielded mixed results.

Limitations and Future Directions

There are several limitations in the current study. First, because we lacked information about whether students actively pursued pre-medicine throughout college, we focused on completion of coursework required for medical programs. This operationalization is indirect and imperfect. It is possible that students of other science majors completed a similar set of coursework, cases that were considered noise in the current study. On the other hand, it is also possible that some students do not or partially complete prerequisite coursework during their undergraduate years, but later complete all required courses in a postbaccalaureate premedical

program. A large number of such programs already existed during the time that data used in the current study was collected (31). Thus, by focusing on students' medical school eligibility in terms of their coursework at their undergraduate institution, the experiences of those that only become eligible later on were not captured.

Second, as we were not provided information about whether students who obtained satisfactory scores on AP exams actually used their AP credits toward their college degree, we assumed that any AP exam score of 3 or higher counted as a fulfillment course. There is the possibility that some students may forfeit their AP credits or take the equivalent college course.

Third, since the collection of data used in the present study, a new version of the MCAT that includes a larger social and behavioral science component has been implemented (32). It is reasonable to assume that requirements by medical schools were also revised to contain psychology and sociology coursework. It will be valuable to examine the effects of these changes. Furthermore, major shifts have taken place with regards to the philosophy underlying the evaluation of medical school applicants. In the past decade, AAMC has endorsed the value of "holistic review," which considers "applicants' experiences, attributes, and academic metrics as well as the value an applicant would contribute to learning, practice, and teaching" (33). As such, academic record and performance is considered alongside many other factors for admission decisions, such as "distance traveled" or cumulative life experiences, and other contextual information for the applicants' accomplishment (34–36). In response to the call for holistic evaluation, a number of medical schools have revised their admissions statements and requirements. For example, the Perelman School of Medicine at the University of Pennsylvania define competencies "not based on specific courses, but rather on the cumulative achievement of knowledge and skills needed to become a physician" (37). The Boston University School of

Medicine emphasize “experiential and personal qualities” in addition to academic rigor in their selection process (38). There are even institutions like Stanford University School of Medicine that explicitly removed any specific prerequisite requirements, and only provide course recommendations instead (39). Since the data used in the current study was collected prior to these changes, it will be important for future research to differently operationalize and examine pre-med intention and persistence.

Finally, as our study relied on archival data, we were limited in the extent to which we could explore underlying mechanisms that explain attrition in pre-medical studies. While the regression analyses revealed certain demographic and academic preparedness factors as predictors, no information about the specific reasons behind students' dropping out was available. However, our study provides a valuable quantitative complement to the prior qualitative body of research that described such reasons.

Conclusion

The present study quantitatively describes the process of attrition as reflected in coursework throughout pre-medical studies in postsecondary institutions. A number of pre-college preparedness factors including socio-economic status, SAT score, and high school GPA as well as grades during college were found to predict continued eligibility for medical studies at at various milestones of relevant coursework.

Structured Disclosures

Acknowledgements: None.

Funding/Support: None.

Ethical approval: Not applicable.

Disclaimer: None.

Previous presentations: None.

References

1. Roese NJ, Summerville A. What We Regret Most... and Why. Personality and Social Psychology Bulletin. 2005;31(9):1273–85.

2. Bastrikin A. Number of College Graduates per Year [Internet]. EducationData. 2019 [cited 2020 Apr 5]. Available from: https://educationdata.org/number-of-college-graduates/

3. Barr DA, Gonzalez ME, Wanat SF. The Leaky Pipeline: Factors Associated With Early Decline in Interest in Premedical Studies Among Underrepresented Minority Undergraduate Students. Academic Medicine. 2008 May;83(5):503.

4. Dyrbye LN, Thomas MR, Power DV, Durning S, Moutier C, Massie FS, et al. Burnout and Serious Thoughts of Dropping Out of Medical School: A Multi-Institutional Study: Academic Medicine. 2010 Jan;85(1):94–102.

5. AAMC. Matriculant Student Questionnaire: 2019 All Schools Summary Report. Association of American Medical Colleges; 2019 p. 26.

6. AAMC. Applicants and Matriculants Data [Internet]. 2018 [cited 2019 Aug 13]. Available from: https://www.aamc.org/data/facts/applicantmatriculant/

7. Williams D. Craking the Code on the “Hidden Curriculum” in the Medical Education Pipeline and its Contribution to Attrition. Journal of Unschooling and Alternative Learning. 2016;10(19):18.

8. Alexander C, Chen E, Grumbach K. How Leaky Is the Health Career Pipeline? Minority Student Achievement in College Gateway Courses. Academic Medicine. 2009 Jun;84(6):797.

9. Fiorentine R. Men, Women, and the Premed Persistence Gap: A Normative Alternatives Approach. American Journal of Sociology. 1987 Mar 1;92(5):1118–39.

10. Fiorentine R, Cole S. Why fewer women become physicians: Explaining the premed persistence gap. Sociol Forum. 1992 Sep 1;7(3):469–96.

11. Lovecchio K, Dundes L. Premed Survival: Understanding the Culling Process in Premedical Undergraduate Education. Academic Medicine. 2002 Jul;77(7):719.

12. Grace MK. Parting ways: Sex-based differences in premedical attrition. Social Science & Medicine. 2019 Jun 1;230:222–33.

13. Chang MJ, Sharkness J, Newman CB, Hurtado S. What Matters in College for Retaining Aspiring Scientists and Engineers. In Denver, CO; 2010. p. 43.

14. Russell ML, Atwater MM. Traveling the road to success: A discourse on persistence throughout the science pipeline with African American students at a predominantly white institution. Journal of Research in Science Teaching. 2005;42(6):691–715.

15. Jacob JA. AAMC Report Examines How to Increase the Pipeline of Black Men Entering Medical School. JAMA. 2015 Dec 1;314(21):2222.

16. Cole D, Espinoza A. Examining the Academic Success of Latino Students in Science Technology Engineering and Mathematics (STEM) Majors. Journal of College Student Development. 2008;49(4):285–300.

17. Carter FE. Predictive factors of premedical student retention and degree completion within a private undergraduate university [Internet] [Dissertation]. Mercer University; 2010 [cited 2019 Jun 29]. Available from: https://search.proquest.com/openview/50480e35b487ed7e3d717a34064340fa/1?pq-origsite=gscholar&cbl=18750&diss=y

18. Barr DA, Matsui J, Wanat SF, Gonzalez ME. Chemistry courses as the turning point for premedical students. Adv in Health Sci Educ. 2010 Mar 1;15(1):45–54.

19. Thurmond VB, Cregler LL. Why students drop out of the pipeline to health professions careers: a follow-up of gifted minority high school students. Academic Medicine : Journal of the Association of American Medical Colleges. 1999;74(4):448–451.

20. Harris RB, Mack MR, Bryant J, Theobald EJ, Freeman S. Reducing achievement gaps in undergraduate general chemistry could lift underrepresented students into a "hyperpersistent zone." Science Advances. 2020 Jun;6(24):eaaz5687.

21. Gonzalez M, Barr DA, Wanat SF. Attrition From Premedical Studies Among Latinas: Case Studies. Hispanic Journal of Behavioral Sciences. 2010 Nov 1;32(4):571–85.

22. Andriole DA, Jeffe DB, Hageman HL, Klingensmith ME, McAlister RP, Whelan AJ. Attrition During Graduate Medical Education: Medical School Perspective. Arch Surg. 2008 Dec 15;143(12):1172.

23. AAMC. How to choose the best premed major [Internet]. Association of American Medical Colleges. 1995 [cited 2020 Apr 5]. Available from: https://students-residents.aamc.org/applying-medical-school/article/how-choose-best-premed-major/

24. Sackett PR, Kuncel NR, Arneson JJ, Cooper SR, Waters SD. Does socioeconomic status explain the relationship between admissions tests and post-secondary academic performance? Psychological bulletin. 2009;135(1):1.

25. Liboff AR, Chopp M. Should the premed requirements in physics be changed? American Journal of Physics. 1979 Apr 1;47(4):331–6.

26. Association of American Medical Colleges. Admission Requirements [Internet]. Association of American Medical Colleges. 2019 [cited 2019 Jul 4]. Available from: https://students-residents.aamc.org/applying-medical-school/applying-medical-school-process/medical-school-admission-requirements/admission-requirements/

27. College Board. AP Credit Policy Search [Internet]. College Board. 2019 [cited 2019 Jun 29]. Available from: https://apstudents.collegeboard.org/getting-credit-placement/search-policies

28. Malgwi CA, Howe MA, Burnaby PA. Influences on Students' Choice of College Major. Journal of Education for Business. 2005 May;80(5):275–82.

29. Arulampalam W, Naylor RA, Smith JP. Dropping out of medical school in the UK: explaining the changes over ten years. Medical Education. 2007;41(4):385–94.

30. Association of American Medical Colleges. Financial Aid [Internet]. Association of American Medical Colleges. 2019 [cited 2019 Jun 29]. Available from: https://students-residents.aamc.org/financial-aid/

31. AAMC. Postbaccalaureate Programs [Internet]. AAMC for Students, Applicants, and Residents. 2020 [cited 2020 Oct 6]. Available from: https://students-residents.aamc.org/postbacc/

32. Frazer NB, Twohig MP. The New Social and Behavioral MCAT Requirements: Inspired Innovation, Missed Opportunity, or Both? Peer Review. 2012;14(4):14–17.

33. AAMC. Holistic Review [Internet]. AAMC. 2020 [cited 2020 Oct 6]. Available from: https://www.aamc.org/services/member-capacity-building/holistic-review

34. Witzburg RA, Sondheimer HM. Holistic Review — Shaping the Medical Profession One Applicant at a Time. New England Journal of Medicine. 2013 Apr 25;368(17):1565–7.

35. Bastedo MN, Bowman NA, Glasener KM, Kelly JL. What are We Talking About When We Talk About Holistic Review? Selective College Admissions and its Effects on Low-SES Students. The Journal of Higher Education. 2018 Sep 3;89(5):782–805.

36. AAMC. Roadmap to excellence: Key concepts for evaluating the impact of medical school holistic admissions. Washington, DC: Association of American Medical Colleges; 2013.

37. Perelman School of Medicine. Admissions Competencies [Internet]. 2020 [cited 2020 Oct 6]. Available from: https://www.med.upenn.edu/admissions/admissions.html

38. Boston University School of Medicine. Selection Factors [Internet]. School of Medicine. 2020 [cited 2020 Oct 6]. Available from: http://www.bumc.bu.edu/busm/admissions/typical-interview-day/

39. Stanford University School of Medicine. Eligibility and Recommendations [Internet]. MD Admissions. 2020 [cited 2020 Oct 6]. Available from: http://med.stanford.edu/md-admissions/how-to-apply/academic-requirements.html

Supporting Information

S1 Table. Variable intercorrelations using sub-sample included in the logistic regression model for Milestone 1.

S2 Table. Variable intercorrelations using sub-sample included in the logistic regression model for Milestone 2.

S3 Table. Variable intercorrelations using sub-sample included in the logistic regression model for Milestone 3.

S4 Table. Variable intercorrelations using sub-sample included in the logistic regression model for Milestone 4.

Fig 1

Click here to access/download;Figure;Fig 1.tiff

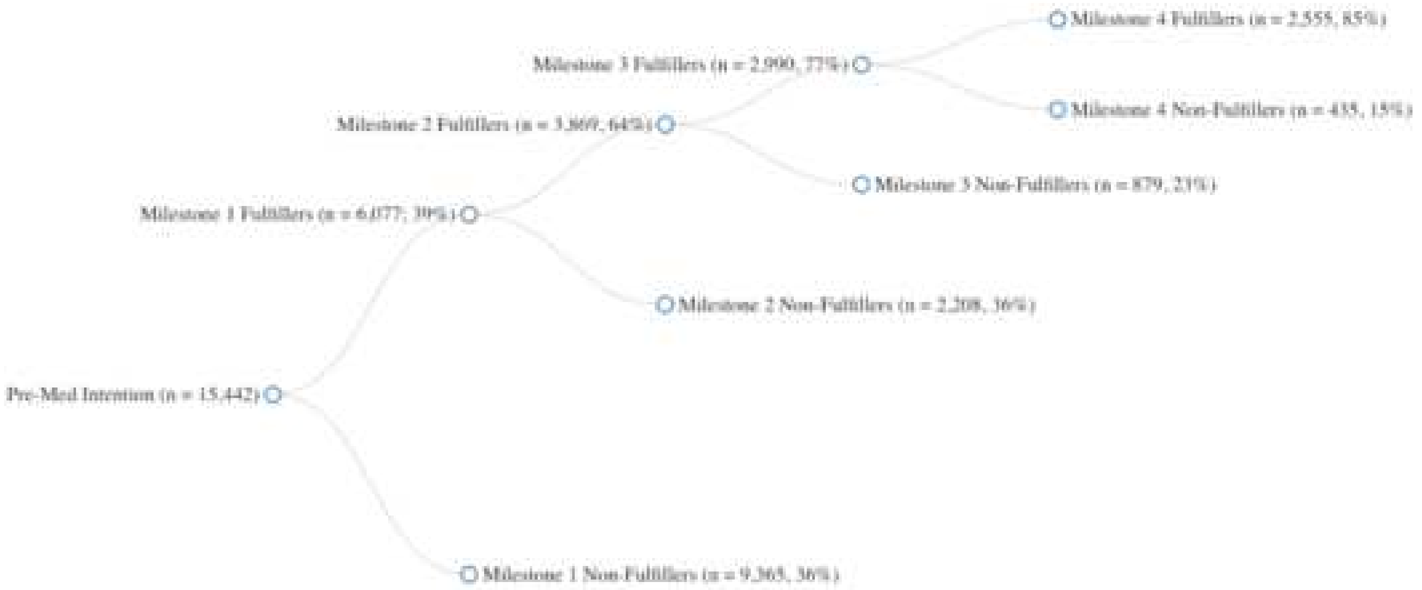

Click here to access/download;Figure;Fig1_201012.tiff

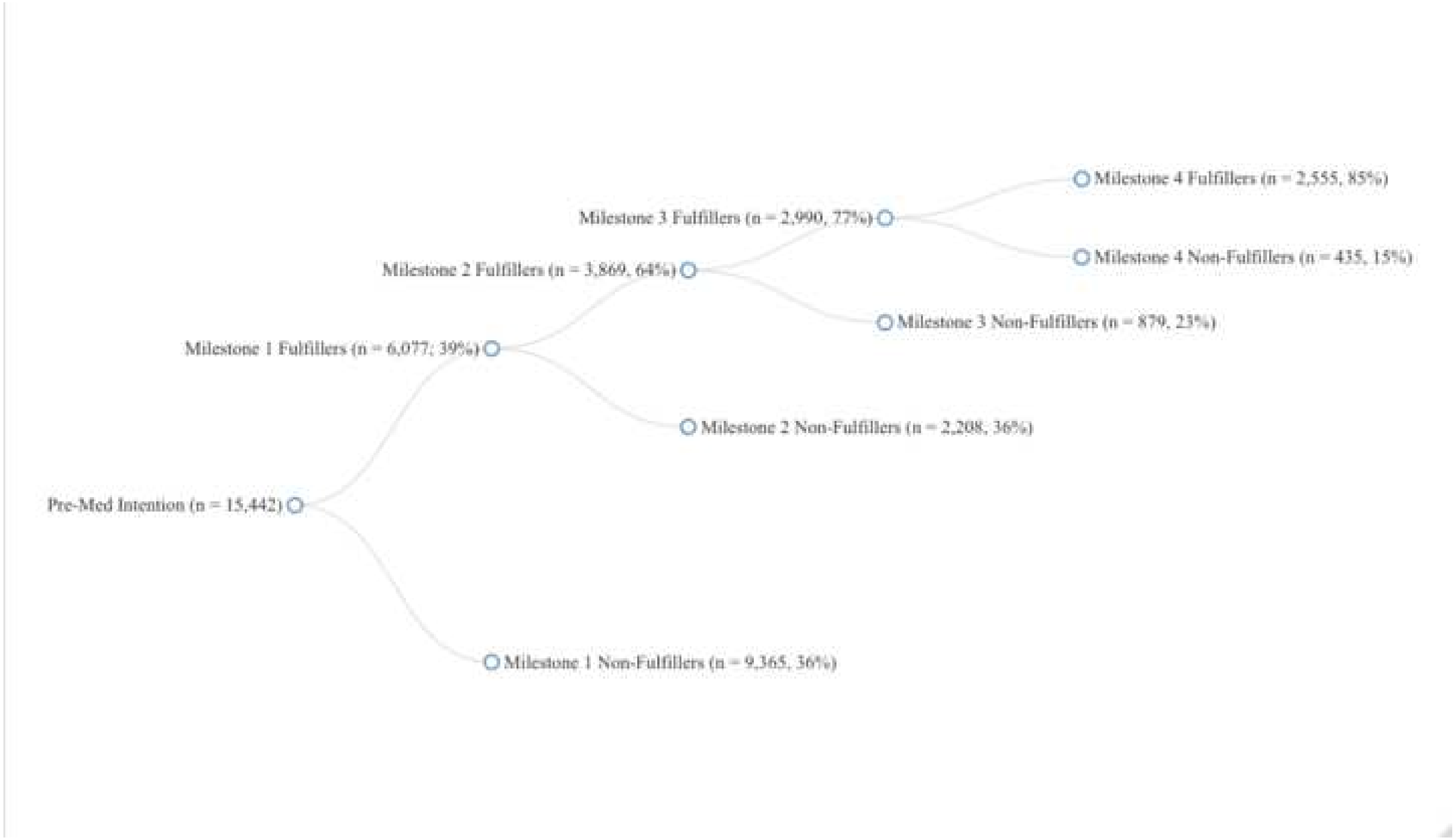

S1 tables a b c d

Click here to access/download
Supporting Information
PLOS_S1_200525.docx

Click here to access/download
Supporting Information
PLOS_S1_200525.docx

Revised Manuscript with Track Changes

The process of attrition in pre-medical studies: A large-scale analysis across 102 schools

Short title: Attrition in pre-medical studies

Charlene Zhang (B.S.)—Corresponding Author
University of Minnesota
75 E River Rd #N496, Minneapolis, MN 55455
zhan5449@umn.edu
Tel: 612-839-2310

Nathan R. Kuncel (Ph.D.)
University of Minnesota
75 E River Rd #N218, Minneapolis, MN 55455
kunce001@umn.edu
Tel: 612-624-1651

Paul R. Sackett (Ph.D.)
University of Minnesota
75 E River Rd #N475, Minneapolis, MN 55455
psackett@umn.edu
Tel: 612-624-9842

Abstract

The important but difficult choice of vocational trajectory often takes place in college, beginning with majoring in a subject and taking relevant coursework. Of all possible disciplines, pre-medical studies are often not a formally defined major but pursued by a substantial proportion of the college population. Understanding students' experiences with pre-med coursework is valuable and understudied, as most research on medical education focuses on the later medical school and residency. We examined the pattern and predictors of attrition at various milestones along the pre-med coursework track during college. Using a College Board dataset, we analyzed a sample of 15,442 students spanning 102 institutions who began their post-secondary education in years between 2006 and 2009. We examined whether students fulfilled the required coursework to remain eligible for medical schools at several milestones: 1) one semester of general chemistry, biology, physics, 2) two semesters of general chemistry, biology, physics, 3) one semester of organic chemistry, and 4) either the second semester of organic chemistry or one semester of biochemistry, and predictors of persistence at each milestone. Only 16.5% of students who intended to major in pre-med graduate college with the required coursework for medical schools. Attrition rates are highest initially but drop as students take more ~~relevant~~ advanced courses. Predictors of persistence include academic preparedness before college (e.g., SAT scores, high school GPA) and college performance (e.g., grades in pre-med courses). Students who perform better academically both in high school and in college courses are more likely to remain eligible for medical school.

Keywords: pre-medical studies, attrition, medical education, coursework, college performance

The Process of Attrition in Pre-Medical Studies: A Large-Scale Analysis Across 102 Schools

All students inevitably face the challenge of choosing their vocational path. For many, the process begins with choosing their college major. This is a difficult but extremely important choice with lasting consequences. Some of the most common regrets of Americans involve their educational and career choices (1). The present study investigates a particular case of career planning—the process through which undergraduate students fulfill prerequisite coursework for medical school. It is no secret that a substantial proportion of high school graduates aspire to a career in the medical field. In fact, health professions and related programs were found to be the second most popular field of study among four-year college students in 2018 (2). However, this group of hopeful young adults often change their mind at one point or another throughout their education (3,4). The journey of pursuit in a medical career involves years of strenuous schooling, not to mention the competitive nature of each step along the trajectory. In 2019, applicants to medical schools submitted a median number of 15 applications and only 42.6% of applicants were accepted to any medical program (5,6). Given the lengthy and strenuous journey of pursuit in a medical career involving years of medical school and residency later and the competitive nature of the field, early years of pre-medical studies in undergraduate is no doubt important. The present study investigates the process through which undergraduate students fulfill prerequisite coursework for medical school. Specifically, we examine rates of attrition at different points in the pre-med curriculum and predictors of continual persistence.

~~A number of studies have examined reasons behind the high rates of attrition in medical education, particularly attrition in medical schools. A systematic review of antecedents~~

~~for dropouts in medical schools and found substantial effects for lower entry qualifications and poor performance in medical school (7).~~

~~While substantial attrition rates have been found in medical schools, past research suggests that attrition begins to occur even earlier.~~ Williams (7) describes the “Pre-Med Syndrome” as the first phase of attrition in the medical education pipeline. ~~However, changing one’s career aspirations from medicine to something else in college and dropping out of medical school likely incur very different consequences. Attrition later in the medical education track (e.g., in medical school) is associated with not only much more wasted time spent for students pursuing medicine, but also wasted financial resources associated with high medical training costs (9). Comparatively, attrition in college is associated with much lower costs in both time and resources invested.~~

Past research has found that upon preparing for medical school application, students are faced with difficult coursework, shattering of unrealistic expectations about what a medical career entails, and harsh admission criteria including high score requirements for the Medical College Assessment Test (MCAT) and college grade point average (GPA). Consistent with this list of challenges, college students report declines in their interest in pre-medical studies (3). Overall, several groups of factors have been linked to attrition in pre-medical studies, namely demographics, academic preparedness, and coursework. We review each of these below.

Certain demographic characteristics have been associated with lower likelihood of persistence in pre-medical studies, specifically being female, and being a member of underrepresented racial and ethnic minority groups (URM). In a longitudinal study following several cohorts of college freshmen in a single college who indicated interest in pre-medical studies (3), women reported a larger decline in interest in continuing medical education than

men, as well as a lower likelihood of applying to medical schools than men. Similar patterns were observed for URM students as compared with non-URM students. However, in another study examining students across six California colleges (8), URM students were found to be almost equal in their likelihood to complete courses required to apply to medical school as non-URM students. On the other hand, demographic characteristics such as having family members who were doctors and higher family income have been found to aid with persistence (3).

Studies have examined whether the gender discrepancy in persistence is coupled with college performance. Fiorentine reported that while males and females with high levels of college performance were equally likely to apply to medical schools, females with low performance were less likely to apply than males with similarly low performance (9). A normative alternatives explanation was proposed such that differences in gender norms provide males with more disincentives to changing their career trajectories when faced with setbacks (10). Consistent with this, Lovecchio and Dundes reported gender to be a moderator for the relationship between performance in organic chemistry courses, with women more likely to alter their career plans as a result of poor performance than men (11). Similarly, pre-medical females construed their own low performance as a sign of poor fit with the medical education track, but not males (12).

The issue of underrepresentation of students from URM groups in medicine and even the broader science, technology, engineering, and mathematics (STEM) field is systemic, beginning with being less prepared in pre-college and college courses (13). Less access to and participation in AP science courses, lack of support and guidance from family and faculty mentors, and financial challenges can all affect whether students from URM persist (14–16).

Additionally, there has been some suggestion that academic preparedness and performance may influence decisions about persistence in pre-medical education. Among factors prior to college, high school GPA was found to significantly predict pre-medical student retention (17). However, Barr and colleagues found no association between SAT scores and persisting interest in pre-medical education (3). In a study by Lovecchio and Dundes, 68% of the former pre-med students surveyed pointed to low grades during college as a major concern for their dropping out (11).

When inquired about specific coursework that deterred students from persisting in their interest in medicine in college, frequently mentioned were low grades obtained in difficult pre-medical “gateway” courses, especially the notorious organic chemistry (3,11,18,19). Further, the discouraging effects of such chemistry course have been found to be especially pronounced for students from URM groups and women (3,18,20).

While this body of research points to demographics, scholastic preparedness, and college performance as predictors of attrition in the pre-medical curriculum, many causes have been derived qualitatively using small-sample interviews and case studies (3,11,12,18,19,21). Among studies that quantitatively and longitudinally examined predictors of medical education attrition, many used multiple cohorts from a single institution (22). A factor that perhaps can partially account for this lack of large-scale, quantitative research on the undergraduate experience of premed students is that pre-medical studies is not a well-defined major in most post-secondary institutions in the United States. Students who are on the pre-med track often major in biological sciences, physical sciences, health sciences, and some even in humanities and social sciences (23). Therefore, it can be difficult to identify students who are in various majors but are in actuality on the premed track. The present study takes an indirect approach. Rather than

attempting to directly identifying the group of pre-med undergraduates, we use all four year of students' coursework data to distinguish those who do not graduate with the basic pre-requisite coursework for medical school from those who do. The combination of this coursework criterion and students' self-reported intentions to pursue a pre-med track before let us reasonably estimate the group of pre-med students.

Furthermore, much of the previous work focused on the singular, final status of persisted versus dropped-out. Thus, the approximately four-year process of pre-medical coursework has been glossed over. The 2019 Matriculating Student Questionnaire (MSQ) administered by the Association of American Medical Colleges (AAMC) reported that while a majority of respondents decided that they wanted to study medicine before college, a substantial percentage (34.8%) decided during their four years of college, most of whom (22.1%) decided during the first two years of college (5). There is much to be gained from examining the patterns of attrition throughout the various stages or milestones of achieving a pre-medical degree and completing medical school prerequisite courses.

Our paper focuses on progress through the science prerequisites for medical school among students stating that pre-med is their intent when they take the SAT. Using data collected by the College Board, we have a sample of 15,442 students from 102 post-secondary institutions across the United States for whom we have a complete record of course-taking in college. Based on the required courses for entry into medical school, we are able to examine which students remain medical school-eligible at various milestones: 1) one semester of general chemistry, biology, and physics, 2) two semesters of general chemistry, biology, and physics, 3) one semester of organic chemistry, and 4) either the second semester of organic chemistry or one semester of biochemistry, as well as predictors of fulfillment at each milestone.

We note that our focus here is on whether or not a student stating an initial pre-med intent completes the academic requirements to be eligible to apply to medical school, not on whether the student does or does not apply to medical school. This is a consequence of using a large dataset which contain rich details on individual course-taking provided by 102 colleges and universities. The data are anonymized, prohibiting inquiry into students plans and choices following graduation. This is clearly a limitation of the study, but we view the access to this rare large-scale data base on medical school eligibility as a worthwhile tradeoff.

Method

Sample

Data from students who began their post-secondary education in academic years between 2006 and 2009 were provided by The College Board. Of the 917,459 individuals whose intended major choice information was available, 170,866 individuals had four years of complete coursework data available and attended a school using a standard semester system. Of those, 153,512 students did not indicate any intention in studying pre-medicine at the time of SAT, 1,912 indicated pre-medical studies as their secondary or tertiary major choice, or indicated pre-medical studies as their first choice major but were not certain of their choice, and 15,442 indicated pre-medicine as their first choice major and were very or fairly certain of their choice. This resulted in our primary sample of 15,442 students spanning 102 institutions.

Measures

Demographics. Gender and race/ethnicity information were provided. Of 15,442 students, 9,852 (63.80%) were female and 5,590 (36.20%) were male. Other than 331 (2.01%) individuals whose race/ethnicity information was missing, 8,130 (52.65%) were White, 2,899 (11.39%) were Asian or Pacific Islander, 1,764 (11.42%) were Black or African American, 1,674

(10.84%) were Hispanic, 67 (.43%) were American Indian or Alaska native, and 597 (3.87%) identified as Other race/ethnicity.

Socioeconomic status (SES). Three SES variables were available: father's education, mother's education, and parental income. Parental income was reported on response options that consisted of several income ranges. A dollar value for parental income is calculated by taking the natural logarithm of the midpoint in each income bracket, thus normalizing the distribution. A equally weighted composite of the three variables were calculated by standardizing each variable individually, summing the three, then standardizing the sum again, following the procedure specified by (24).

Intended major choice. Students indicated their intended major choice from a list of 368 different majors at the time they took the SAT.

~~**Degree goal (DEGGOAL).** At the time that students took the SAT, they indicated the highest level of education that they intend to pursue. Responses included: 1) specialized training or certificate program; 2) two-year associate of arts or sciences degree; 3) bachelor's degree; 4) master's degree; and 5) doctoral or related degree.~~

High school GPA (hsGPA). Self-reported GPA was used.

SAT scores. Three SAT scores were provided based on the three subsections: Verbal/Critical Reading (SATV), Writing (SATW), and Math (SATM), with possible scores ranging from 200 to 800. A composite SAT score (SATC) was calculated by summing the three subsection scores.

AP courses. Data were provided on whether students took any AP courses and their grades on the corresponding AP exams, ranging from 1 to 5. The ones relevant to the pre-medicine curriculum were included: biology, chemistry, calculus AB, calculus BC, english

language and composition, english literature and composition, physics B, physics C: electricity and magnetism, physics C: mechanics, and statistics.

College coursework. For all college courses that are taken by each student, information was provided about the course name, the year and semester in which the course was taken, the content area in which courses fell, and the grade obtained.

Procedure

Students' eligibility to apply to medical schools (persistence in studying medicine) was operationalized as whether they fulfill the standard coursework required by most medical programs. To determine medical school prerequisites, we tallied definitions used by prior research (8,9,25), admission requirements specified by the Association of American Medical Colleges (AAMC), and course requirements of 105 medical schools across the United States in 2013.

AAMC suggested general prerequisites to be one year of Biology, one year of Physics, and two years of Chemistry that includes Organic Chemistry courses (26). Some medical programs also allowed one biochemistry course to substitute for the second organic chemistry course. Therefore, we operationalized fulfillment of medical school prerequisites to include one year (two semesters) of biology, physics, general chemistry, and either one year of organic chemistry or one semester of organic chemistry with one semester of biochemistry.

The number of courses in each subject was counted to indicate continued eligibility for medical school at a number of milestones during coursework progression. For a course to be included, a numerical grade of 0.7 or higher on a scale of 0 to 4 needed to be achieved, as it is the lowest passing grade.

Further, many students receive college credit for achieving satisfactory grades on AP exams. AP exam scores of 3 or above were accepted by most schools as course credits (27), and were counted toward prerequisite fulfillment.

Analyses

Descriptive statistics were calculated to examine differences between fulfillers of medical school course pre-requisites and non-fulfillers.

Subsequently, logistic regression analyses at several important coursework milestones were performed to examine the effects of demographics, academic preparedness, and college course performance on course fulfillment at each stage.

Results

Rates of Prerequisite Fulfillment

Overall, among the 15,442 students who indicated initial pre-med interests, 2,555 (16.5%) completed the full set of medical school prerequisites, while a small proportion (7.7%) never completed any chemistry, biology, or physics courses. As shown in Fig 1, the process of dropping out is examined at four major milestones, namely

1) whether students ~~took~~ passed one semester of general chemistry, biology, and physics (39 % did so);
2) among students who fulfilled milestone 1, whether they ~~took~~ passed a second semester of general chemistry, biology, and physics (64% did so);
3) among students who fulfilled milestone 2, whether they ~~took~~ passed one semester of organic chemistry (77% did so);

4) among students who fulfilled milestone 3, whether they ~~took~~ passed a second semester of organic chemistry or a semester of biochemistry, thereby satisfying the entire fulfillment requirement (85% did so).

Fig 1. Pattern of Continuing Fulfillment of Pre-Med Coursework at Various Milestones. Pre-Med Intention = students who indicated intentions of majoring in pre-medicine at the time of taking SAT, Milestone 1 = whether students took a first semester of general chemistry, biology, and physics, Milestone 2 = whether students who completed Milestone1 took a second semester of general chemistry, biology, and physics, Milestone 3 = whether students who completed Milestone2 took a first semester of organic chemistry, Milestone 4 (full fulfillment) = whether students who completed Milestone3 took a second semester of organic chemistry or a semester of biochemistry

Formatted: Font: Bold

Proportions of fulfillers at each milestone were also calculated for each of the 102 institutions individually. Across all institutions, an average of 34% of students fulfilled milestone 1 (SD = 19%), 23% fulfilled milestone 2 (SD = 16%), 19% fulfilled milestone 3 (SD = 16%), and 17% fulfilled milestone 4 (SD = 16%).

To allow comparisons, the number of students who fulfilled prerequisites for medical schools was also tallied for the group of 1,912 students who indicated some intention in studying pre-medicine but were not certain, and the group of 153,512 students who had no intention of studying pre-medicine. 267 (14.0%) fulfilled the full set of medical school prerequisite coursework in the former group and 2,633 (3.9%) fulfilled it in the latter group.

Comparison between Fulfillers and Non-Fulfillers of Medical School Prerequisites

Demographic information of the 2,555 fulfillers and 12,887 non-fulfillers of medical school prerequisites can be found in Table 1. A larger proportion of males (21%) who reported pre-med intentions fulfilled the prerequisites than females (14%).

Table 1. Sample Size and Percentage of Prerequisite Fulfillers at Each Milestone by Gender and Race

		Percentage Within Group that Fulfilled Milestone			
Group	n	M1	M2	M3	M4
Overall	15,442	39.35%	25.06%	19.36%	16.55%
Gender					
Male	5,590	45.65%	30.41%	23.94%	21.13%
Female	9,852	35.78%	22.02%	16.77%	13.95%
Race					
White	8,130	38.18%	24.24%	19.11%	16.30%
Asian	2,899	50.85%	32.63%	26.11%	23.28%
Black	1,764	30.05%	17.74%	12.24%	9.35%
Hispanic	1,674	33.63%	22.70%	15.11%	12.54%
American Indian	67	22.39%	16.42%	11.94%	8.96%
Other	597	44.39%	28.48%	22.95%	19.60%
Race x Gender					
White Male	3,080	43.67%	29.16%	23.54%	20.65%
White Female	5,050	34.83%	21.25%	16.42%	13.64%
Asian Male~~/~~	1,205	53.69%	35.02%	27.88%	26.14%
Asian~~/~~ Female	1,694	48.82%	30.93%	24.85%	21.25%
Black Male	353	39.09%	24.93%	17.00%	12.75%
Black Female	1,411	27.78%	15.95%	11.06%	8.50%
Hispanic Male	586	41.30%	30.72%	21.67%	18.09%
Hispanic Female	1,088	29.50%	18.38%	11.58%	9.56%
American Indian Male	24	16.67%	12.50%	8.33%	8.33%
American Indian Female	43	25.58%	18.60%	13.95%	9.30%
Other Male	213	53.99%	32.86%	25.35%	22.54%
Other Female	384	39.06%	26.04%	21.61%	17.97%

Notes. M1 = Milestone 1, including a first semester of general chemistry, biology, and physics, M2 = Milestone 2, including two semesters of general chemistry, biology, and physics, M3 = Milestone 3, including Milestone2 as well as a first semester of organic chemistry, M4 = Milestone 4 (full fulfillment), including Milestone 3 as well as a second semester of organic chemistry or a semester of biochemistry. American Indian = American Indian or Alaska Native, Asian = Asian, Asian American, or Pacific Islander, Black = Black or African American, Hispanic = Mexican or Mexican American, Puerto Rican, or Other Hispanic, Latino, or Latin American, Other = Other minorities.

Formatted: Tab stops: Not at 0.16"

Formatted: Font color: Auto

With regards to race/ethnicity, rates of fulfilling prerequisites among those who had intention of pursuing pre-medical studies were ~~23%~~the highest for Asians (23%), ~~20%~~ followed

by the ~~for~~ Other Minority group (20%), then ~~16% for~~ Whites (16%) and ~~, 13% for~~ Hispanics (13%), and ~~9%~~the lowest for Blacks (9%).

There were meaningful differences between fulfillers and non-fulfillers on a variety of variables, both prior to college and throughout college (see Table 2). Fulfillers scored higher than non-fulfillers on the SAT-Combined (*d* = .44), with the largest difference in the Math section (*d* = .51). Fulfillers also reported higher GPA, both in high school (*d* = .33), and in all four years of college (average *d* = .37). Higher socioeconomic status (*d* = .18) ~~and degree goal (*d* = .10)~~ ~~were~~was reported by fulfillers than non-fulfillers. In cases where AP grades were available, fulfillers obtained higher AP scores than non-fulfillers, including AP Biology (*d* = .52), AP Chemistry (*d* = .38), and AP Physics B and C (average *d* = .50).

Table 2 Descriptive Statistics for Continuous Variables for Fulfillers and Non-Fulfillers

	Fulfillers			Non-Fulfillers			
Variables	N	Mean	SD	N	Mean	SD	d
SAT~~V~~-Verbal	2,516	601.60	84.45	12,748	572.45	87.06	.34
SAT-Math~~M~~	2,516	636.87	81.81	12,748	591.94	89.32	.51
SAT-Writing~~W~~	2,508	599.53	86.62	12,727	571.09	88.67	.32
SAT-Composite~~C~~	2,508	1838.05	222.62	12,727	1735.54	236.47	.44
~~HSGPA~~High School GPA	2,545	3.96	.32	12,786	3.83	.39	.33
~~CGPA1~~	~~2,552~~	~~3.39~~	~~.47~~	~~12,836~~	~~3.07~~	~~.60~~	~~.54~~
~~CGPA2~~	~~2,548~~	~~3.33~~	~~.53~~	~~12,706~~	~~3.11~~	~~.61~~	~~.37~~
~~CGPA3~~	~~2,548~~	~~3.37~~	~~.56~~	~~12,634~~	~~3.17~~	~~.64~~	~~.31~~
~~CGPA4~~	~~2,542~~	~~3.39~~	~~.59~~	~~12,789~~	~~3.21~~	~~.70~~	~~.27~~
SES	2,471	.34	.94	12,504	.17	.97	.18
~~DEGGOAL~~	~~2,452~~	~~8.87~~	~~.74~~	~~12,123~~	~~8.77~~	~~.97~~	~~.10~~
AP grades							
AP Biol	1,167	3.75	1.20	4,204	3.06	1.39	.52
AP Chem	817	3.18	1.29	2,376	2.68	1.35	.38
AP Phys B	303	3.14	1.24	959	2.75	1.29	.31
AP Phys E	70	3.73	1.24	135	2.88	1.42	.62
AP Phys M	146	3.74	1.11	346	3.01	1.33	.57

Formatted: Left

Formatted Table

Number of courses taken							
Biochem N	2,555	.66	.86	12,887	.20	.51	.32~~80~~
Biol N	2,555	5.99	3.00	12,887	2.56	2.51	.65~~1.32~~
GenChem N	2,555	2.81	1.34	12,887	1.54	1.28	.53~~98~~
OrgChem N	2,555	2.16	.93	12,887	.44	.80	.83~~2.09~~
Phys N	2,555	2.35	.79	12,887	.72	1.24	.77~~1.38~~
College grades							
Biochem GR	1,178	3.15	.83	2,020	2.88	.94	.30
Biol GR	2,547	3.16	.68	9,754	2.71	.90	.52
GenChem GR	2,545	3.16	.69	9,382	2.66	.95	.56
OrgChem GR	2,547	2.90	.85	3,746	2.58	.96	.35
Phys GR	2,524	3.20	.75	4,599	2.88	.94	.37

Note. Fulfillment = two semesters of general chemistry, biology, physics, and organic chemistry or one semester of organic chemistry with one semester of biochemistry, d = Cliff's δ for number of courses and Cohen's d for all other variables, ~~SATV = SAT Verbal/Critical Reading, SATM = SAT Mathematics, SATW = SAT Writing, SATC = SAT Composite, HSGPA = self-reported high school GPA, CGPA1 = first-year college GPA, CGPA2 = second-year college GPA, CGPA3 = third-year college GPA, CGPA4 = fourth-year college GPA,~~ SES = socio-economic status, ~~DEGGOAL = degree goal,~~ Biol = Biology, Chem = Chemistry, Phys B = Physics, Phys E = Physics C Electricity & Magnetism, Phys M = Physics C Mechanics, Biochem = Biochemistry, GenChem = General Chemistry, OrgChem = Organic Chemistry~~, N = number of courses taken in college, GR = grade~~.

When college performance is broken down specifically by course, fulfillers were found to have taken a greater number of medical school prerequisite courses (average *d* = 1.31) as coursework definitionally distinguishes fulfillers from non-fulfillers. They were also found to have ~~as well as have~~ performed better in ~~them~~ these courses (average *d* = .42).

Logistic Regression Models for Predicting Fulfillment Progression

Logistic regression analyses were performed for the four fulfillment milestones. Predictors included gender, ethnicity, the composite SAT score, high school GPA, SES, ~~degree goal,~~ and mean college pre-med course grades when applicable. Continuous variables—SAT, GPA, SES, and course grades—were standardized to aid the comparison between regression

Formatted: Left

Formatted Table

Formatted: Left

Formatted Table

coefficients. Variable intercorrelations with each subset of the sample can be found in S1a, b, c, dS1, 2, 3, and 4 tables in the supplement. The progression of logistic regression models can be found in Table 3. The same models with individual prior course grades as predictors were also tested and yielded similar results.

Table 3 reports the logistic regression coefficients as well as the effect sizes in odds ratios (OR). To aid the interpretation of these results, we also computed predicted likelihoods based on the regression weights. To compute fulfillment likelihoods of various demographic groups, we plugged in average values of all other predictors. To compute the continuous variables' effects on fulfillment likelihoods, we plugged in the reference groups (i.e., females for gender, White for race), average values of all other predictors, and the average and 1 standard deviation (SD) above average values of the predictor of interest.

Table 3

Summary of Logistic Regression Analysis For Various Milestones Towards Pre-Med Course Fulfillment

	Milestone 1			Milestone 2			Milestone 3			Full Fulfillment (Milestone 4)		
Predictor	*B*	e^B	*SE (B)*	*B*	e^B	*SE (B)*	*B*	e^B	*SE (B)*	*B*	e^B	*SE (B)*
Intercept	-.70**	.50	.03	.42**	1.52	.05	1.25**	3.48	.07	1.28**	3.59	.08
Gender: Male	.36**	1.44	.04	.19**	1.21	.06	.14	1.15	.08	.17	1.18	.10
Race: Black	.04	1.05	.06	.14	1.15	.11	-.15	.86	.15	-.30	.74	.18
Race: Asian	.50**	1.65	.05	.07	1.07	.07	.02	1.02	.10	-.07	.93	.11
Race: Hispanic	.04	1.04	.06	.37**	1.44	.11	-.44**	.64	.13	.16	1.17	.18
SATC	.21**	1.23	.02	-.05	.95	.04	.28**	1.33	.05	-.13*	.88	.06
HSGPA	.24**	1.28	.02	-.03	.97	.03	.15**	1.17	.04	.03	1.03	.05
SES	.04*	1.04	.02	-.05	.95	.03	-.05	.95	.04	.01	1.01	.05
~~DEGGOAL~~	~~.04~~	~~1.04~~	~~.02~~	~~.03~~	~~1.03~~	~~.03~~	~~.09~~	~~1.09~~	~~.05~~	~~.09~~	~~1.10~~	~~.06~~
Grades				.44**	1.56	.03	.07	1.07	.05	.19*	1.21	.07
OChem										-.01	.99	.07
df	13,786			5,404			3,419			2,610		
pseudo R^2	.07			.06			.05			.02		
AIC	17,768			6,903			3,603			2,704		

Note. Reference group for Gender was Female. Reference group for Race was White. Milestone1 = whether students took a first semester of general chemistry, biology, and physics, Milestone2 = whether students who completed Milestone1 took a second semester of general chemistry, biology, and physics, Milestone3 = whether students who completed Milestone2 took a first semester of organic chemistry, Full Fulfillment (Milestone4) = whether students who completed Milestone3 took a second semester of organic chemistry or a semester of biochemistry, Race: Black = Black or African American, Race: Asian = Asian, Asian American, or Pacific Islander, Race: Hispanic: Mexican or Mexican American, Puerto Rican, or Other Hispanic, Latino, or Latin American, SATC = standardized SAT composite, HSGPA = standardized self-reported high school GPA, SES = standardized socio-economic status, ~~DEGGOAL = degree goal,~~ Grades = standardized average grade of all prior courses in general chemistry, biology, and physics, OChem = standardized grade in first-semester organic chemistry, df = residual degrees of freedom, pseudo R^2 = Nagelkerke's R^2 .

**p* < .05

***p* < .001

Formatted Table

Formatted: Width: 11", Height: 8.5"

Model 1 examined whether students took first-semester general chemistry, biology, and physics courses with demographic and pre-college predictors (milestone 1). The predicted likelihood of males fulfilling milestone 1 was 8.50% higher than that of females (odds ratio (OR) = 1.44), and t~~T~~he likelihood of Asian students completing the milestone was 11.97% higher than that of White students (OR = 1.65), net of all other predictors.~~The likelihood of completing this first milestone was 1.44 times greater for males than females, and 1.62 times higher for Asian than White students.~~ Further, controlling for all other predictors, ~~students with higher overall scores on SAT (odds ratio (OR) = 1.22), higher high school GPA (OR= 1.28), and higher socioeconomic status (OR = 1.05) were more likely to fulfill these prerequisites~~the predicted likelihood of fulfilling milestone 1 increased by 4.82% with every 1 SD increase in SAT score (OR = 1.23), 5.63% with every 1SD increase in high school GPA (OR = 1.28), and .98% with every 1SD increase in SES (OR = 1.04).

Model 2 examined whether students who completed a first semester of prerequisites proceeded to complete a second semester of general chemistry, biology, and physics (milestone 2), using not only demographic and pre-college predictors, but also the mean of first-semester general chemistry, biology, and physics grades. Among students who had fulfilled milestone 1, the predicted likelihood of males fulfilling milestone 2 was 4.48% higher than that of females (OR = 1.21), and the likelihood of Hispanic students was 8.34% higher than that of White females (OR = 1.44) when holding all other predictors constant. ~~Being male (OR = 1.21) and being Hispanic (OR= 1.44) were both associated with higher likelihood of taking a second semester of pre-med courses.~~ Further, ~~one standard deviation~~1SD increase in average first-semester course grades ~~was related with a 1.58 times greater likelihood~~improved the likelihood

of students fulfilling milestone 2 by 10.04% to complete these coursesnet of all other predictors (OR = 1.56).

Subsequently, model 3 used the same set of predictors with course grades computed as the average of grades across both semesters of general chemistry, biology, and physics to determine whether students who have fulfilled milestone 2 took any organic chemistry courses (milestone 3). Among students who had completed milestone 2, the likelihood of Hispanic students taking and passing an organic chemistry course was 8.58% lower than that of White females when holding all other predictors constant at average (OR = .64). The likelihood of fulfilling milestone 3 also increased by 4.51% with every 1SD increase in SAT score (OR = 1.33) and by 2.54% with every 1SD increase in high school GPA (OR = 1.17), net of all other variables. Hispanic students were only .61 times as likely to take organic chemistry as White students. Both higher SAT scores (OR = 1.33) and higher high school GPA (OR = 1.16) were associated with greater likelihood of completing an organic chemistry course.

Finally, Model 4 used the same previous set of predictors along with the first organic chemistry grade to predict whether students took a second organic chemistry course or a biochemistry course, thereby fulfilling all science coursework prerequisites for medical school, conditional on having completed all required courses thus far (milestone 4). There were no statistically significant difference in fulfillment likelihood between gender and racial majority and minority groups.Males were 1.23 times more likely to complete this last step than females. However, Controlling for all other predictors, 1SD higher increase SAT scores decreased predicted likelihood of prerequisite completion by 2.24% (OR = .887), and 1SD were associated with lower likelihood. Lastly, one standard deviation increase in average coursework grades was

accompanied by a 1.18 times increase in likelihoodincreased likelihood by 3.09% (OR = 1.21) of prerequisite completion.

To explore the potential demographic variables' interactions in predicting coursework fulfillment likelihoods, the same four models were also tested including the interaction terms between gender and race dummy variables. The only statistically significant and meaningful interaction was between being male and being Asian for fulfilling coursework milestone 2 (OR = .71, $p < .001$) and milestone 3 (OR = .69, $p < .001$). In other words, holding all other predictors constant at their average values, while the predicted likelihood of White males fulfilling milestone 2 exceeds that of White females by 8.67%, the difference in likelihood between Asian males and females is only 2.14%. Similarly, the difference between White males' and females' predicted likelihood for completing milestone 3 is 7.81%, while that between Asian males and females is 1.53%.

Additional logistic regression models with various interactions terms between demographic dummy variable (i.e., gender and race) and the continuous predictors (i.e., SAT, high school GPA, SES, and grades) were also tested individually. For predicting milestones 1, 2, and 3, there was a significant interaction between gender and SAT score such that the difference in fulfillment likelihood between males and females (in favor of males) was reduced as SAT score increased (OR = .88, .84, and .88, respectively). A similar effect was found in the White–Asian comparison for milestones 1 and 2, such that the higher fulfillment likelihood of Asian students than White students was reduced with higher SAT scores (OR = .83 and .84, respectively). In addition, for predicting fulfillment of milestone 3, the higher likelihood of Asian than White students was reduced as average college course grade increased (OR = .88). Lastly,

the higher likelihood of Asian students fulfilling milestone 4 than White students was enhanced with higher high school GPA (OR = 1.31).

Discussion

The current study examines the pre-medical coursework fulfillment patterns of the group of students who indicated intentions of studying pre-medicine prior to entering college. Only 16.5% of the students graduated with the coursework required by most medical schools. Attrition is highest at early stages and levels off as students commit to the medical education track by taking more of the required courses. Previous studies that found that former pre-med students often mentioned a “distaste for the large pre-med classes” and the highly competitive environment (3), and a change in interest as a result of exposure to other subjects (3,12,19). Thus, while attrition rates in the later college years are comparatively lower and may be attributed to challenging coursework, the initial high attrition may reflect students adjusting their expectations about medicine while discovering interest in non-medical disciplines. This is also consistent with earlier findings that students change their majors often due to interest in and positive perceptions of new major more than negative factors about the old major (28). Given the low acceptance rates into medical schools (6) and the attrition rates in medical schools (4,29), this early change in education track may actually prevent additional personal resources from being wasted in the process of applying to medical programs or institutional resources from being wasted when students drop out of medical programs.

Although a much higher percentage of intended pre-med students completed the full set of medical school prerequisite courses and ended up eligible for medical schools (16.5%) than students with no initial intent (3.9%), the absolute number of the latter group (2,633) was comparable with that of the former group (2,555). The 2019 MSQ by the AAMC reported that of

the medical school matriculants, 55.3% had decided that they wanted to study medicine prior to entering college and 34.8% did so during college (6). When interpreted in light of the present findings, it is evident that a higher percentage of the students who completed medical school prerequisite coursework with initial intent is accepted than without initial intent.

Predictors of Pre-med Persistence

Similar to previous findings on the association between gender and attrition (3,10) and consistent with the normative alternatives approach to explaining the persistence gap (9), being male was linked with a significantly higher likelihood of persisting in a pre-medical education at nearly all stages throughout college. In other words, women's lower likelihood to persist in medicine may be construed as a higher likelihood to accept alternative career choices.

With regards to ethnic and racial identities, while previous investigations suggest that ethnic and racial minority students are less likely to persist (3,8), results of the current study were less consistent. For Asian students, the odds of fulfilling the first semester of coursework were more likely than those of White students, with persistence likelihood decreasing slightly throughout the later milestones but never significantly lower than those of White students. The odds of Hispanic students fulfilling the first year of coursework were higher than those of White students, but the odds of their completing organic chemistry were lower. Lastly, African American students did not differ from White students in their likelihood of persistence at any point after controlling for socio-economic status, SAT, and grades in high school and college..

Further, students who fulfilled all required coursework reported higher SES. Although SES predicted completion of the first semester of required coursework, it did not predict persistence at any of the later milestones. Thus, the advantage of coming from a family with higher SES found by (3) seems to wear off early on during college. However, its link with

persistence in a medical education is likely to strengthen when students decide whether or not to attend medical school, as a $200,000 to $300,000 cost for medical school is no small expense (30).

Consistent with the reputation of a degree in pre-medical studies for being cognitively intensive and challenging, coursework fulfillers entered college with higher scores on all components of the SAT as well as higher high school GPA. Continuing with this advantage, college GPA's of students who eventually fulfilled all required coursework were higher than the GPA's of those who did not for all four years. However, the differences declined over time, with the GPA difference in the fourth year of college being half as large as the GPA difference in the first year of college. This may be an indirect reflection of the high levels of difficulty in pre-medical courses compared with other courses. In terms of college performance, coursework fulfillers both by definition completed a larger number of courses in relevant science subjects and obtained higher grades in them than non-fulfillers.

When examining the predictive validities of academic preparedness measured by variables prior to college (SAT and high school GPA) and college performance (course grades), an interesting pattern is observed. For predicting completion of the first semester of coursework in the absence of any college grades, higher academic preparedness was associated with a greater likelihood of completion. However, the predictive validities of such more distal, pre-college factors were overtaken by that of the more proximal college grades. The exception is whether students complete the first organic chemistry, for which academic preparedness rather than college grades was predictive. This may be due to the notoriously difficult organic chemistry being overwhelmingly identified as culprit in the leaky pipeline (3,11). Students might be extra cautious in deciding whether they would be able to succeed in the course and resort to

information about their academic effectiveness over a longer period of their lives from the past rather than grades from the recent one or two years to make the decision. In a recent study examining persistence in undergraduate STEM courses, it was found that grades in the first general chemistry course was related with subsequent persistence in STEM more strongly among underrepresented individuals than among well-represented students (20). Our examination of such interactions between demographic group and other predictors (e.g., academic preparedness before college, grades in college) yielded mixed results.

Limitations and Future Directions

There are several limitations in the current study. First, because we lacked information about whether students actively pursued pre-medicine throughout college, we focused on completion of coursework required for medical programs. This operationalization is indirect and imperfect. It is possible that students of other science majors completed a similar set of coursework, cases that were considered noise in the current study. On the other hand, it is also possible that some students do not or partially complete prerequisite coursework during their undergraduate years, but later complete all required courses in a postbaccalaureate premedical program. A large number of such programs already existed during the time that data used in the current study was collected (31). Thus, by focusing on students' medical school eligibility in terms of their coursework at their undergraduate institution, the experiences of those that only become eligible later on were not captured.

Second, as we were not provided information about whether students who obtained satisfactory scores on AP exams actually used their AP credits toward their college degree, we assumed that any AP exam score of 3 or higher counted as a fulfillment course. There is the possibility that some students may forfeit their AP credits or take the equivalent college course.

Third, since the collection of data used in the present study, a new version of the MCAT that includes a larger social and behavioral science component has been implemented (32). It is reasonable to assume that requirements by medical schools were also revised to contain psychology and sociology coursework. It will be valuable to examine the effects of these changes. Furthermore, major shifts have taken place with regards to the philosophy underlying the evaluation of medical school applicants. In the past decade, AAMC has endorsed the value of "holistic review," which considers "applicants' experiences, attributes, and academic metrics as well as the value an applicant would contribute to learning, practice, and teaching" (33). As such, academic record and performance is considered alongside many other factors for admission decisions, such as "distance traveled" or cumulative life experiences, and other contextual information for the applicants' accomplishment (34–36). In response to the call for holistic evaluation, a number of medical schools have revised their admissions statements and requirements. For example, the Perelman School of Medicine at the University of Pennsylvania define competencies "not based on specific courses, but rather on the cumulative achievement of knowledge and skills needed to become a physician" (37). The Boston University School of Medicine emphasize "experiential and personal qualities" in addition to academic rigor in their selection process (38). There are even institutions like Stanford University School of Medicine that explicitly removed any specific prerequisite requirements, and only provide course recommendations instead (39). Since the data used in the current study was collected prior to these changes, it will be important for future research to differently operationalize and examine pre-med intention and persistence.

Finally, as our study relied on archival data, we were limited in the extent to which we could explore underlying mechanisms that explain attrition in pre-medical studies. While the

regression analyses revealed certain demographic and academic preparedness factors as predictors, no information about the specific reasons behind students' dropping out was available. However, our study provides a valuable quantitative complement to the prior qualitative body of research that described such reasons.

Conclusion

The present study quantitatively describes the process of attrition as reflected in coursework throughout pre-medical studies in postsecondary institutions. While interventions have been proposed to ameliorate the attrition problem in college, such as improving the undergraduate advising system (3) and reforming the pre-medical curriculum (42,43), our results question the need of any intervention in the very early stages of the medical education track.A number of pre-college preparedness factors including socio-economic status, SAT score, and high school GPA as well as grades during college were found to predict continued eligibility for medical studies at at various milestones of relevant coursework.

Structured Disclosures

Acknowledgements: None.

Funding/Support: None.

Ethical approval: Not applicable.

Disclaimer: None.

Previous presentations: None.

References

1. Roese NJ, Summerville A. What We Regret Most... and Why. Personality and Social Psychology Bulletin. 2005;31(9):1273–85.

2. Bastrikin A. Number of College Graduates per Year [Internet]. EducationData. 2019 [cited 2020 Apr 5]. Available from: https://educationdata.org/number-of-college-graduates/

3. Barr DA, Gonzalez ME, Wanat SF. The Leaky Pipeline: Factors Associated With Early Decline in Interest in Premedical Studies Among Underrepresented Minority Undergraduate Students. Academic Medicine. 2008 May;83(5):503.

4. Dyrbye LN, Thomas MR, Power DV, Durning S, Moutier C, Massie FS, et al. Burnout and Serious Thoughts of Dropping Out of Medical School: A Multi-Institutional Study: Academic Medicine. 2010 Jan;85(1):94–102.

5. AAMC. Matriculant Student Questionnaire: 2019 All Schools Summary Report. Association of American Medical Colleges; 2019 p. 26.

6. AAMC. Applicants and Matriculants Data [Internet]. 2018 [cited 2019 Aug 13]. Available from: https://www.aamc.org/data/facts/applicantmatriculant/

7. Williams D. Craking the Code on the “Hidden Curriculum” in the Medical Education Pipeline and its Contribution to Attrition. Journal of Unschooling and Alternative Learning. 2016;10(19):18.

8. Alexander C, Chen E, Grumbach K. How Leaky Is the Health Career Pipeline? Minority Student Achievement in College Gateway Courses. Academic Medicine. 2009 Jun;84(6):797.

9. Fiorentine R. Men, Women, and the Premed Persistence Gap: A Normative Alternatives Approach. American Journal of Sociology. 1987 Mar 1;92(5):1118–39.

10. Fiorentine R, Cole S. Why fewer women become physicians: Explaining the premed persistence gap. Sociol Forum. 1992 Sep 1;7(3):469–96.

11. Lovecchio K, Dundes L. Premed Survival: Understanding the Culling Process in Premedical Undergraduate Education. Academic Medicine. 2002 Jul;77(7):719.

12. Grace MK. Parting ways: Sex-based differences in premedical attrition. Social Science & Medicine. 2019 Jun 1;230:222–33.

13. Chang MJ, Sharkness J, Newman CB, Hurtado S. What Matters in College for Retaining Aspiring Scientists and Engineers. In Denver, CO; 2010. p. 43.

14. Russell ML, Atwater MM. Traveling the road to success: A discourse on persistence throughout the science pipeline with African American students at a predominantly white institution. Journal of Research in Science Teaching. 2005;42(6):691–715.

15. Jacob JA. AAMC Report Examines How to Increase the Pipeline of Black Men Entering Medical School. JAMA. 2015 Dec 1;314(21):2222.

16. Cole D, Espinoza A. Examining the Academic Success of Latino Students in Science Technology Engineering and Mathematics (STEM) Majors. Journal of College Student Development. 2008;49(4):285–300.

17. Carter FE. Predictive factors of premedical student retention and degree completion within a private undergraduate university [Internet] [Dissertation]. Mercer University; 2010 [cited 2019 Jun 29]. Available from: https://search.proquest.com/openview/50480e35b487ed7e3d717a34064340fa/1?pq-origsite=gscholar&cbl=18750&diss=y

18. Barr DA, Matsui J, Wanat SF, Gonzalez ME. Chemistry courses as the turning point for premedical students. Adv in Health Sci Educ. 2010 Mar 1;15(1):45–54.

19. Thurmond VB, Cregler LL. Why students drop out of the pipeline to health professions careers: a follow-up of gifted minority high school students. Academic Medicine : Journal of the Association of American Medical Colleges. 1999;74(4):448–451.

20. Harris RB, Mack MR, Bryant J, Theobald EJ, Freeman S. Reducing achievement gaps in undergraduate general chemistry could lift underrepresented students into a “hyperpersistent zone.” Science Advances. 2020 Jun;6(24):eaaz5687.

21. Gonzalez M, Barr DA, Wanat SF. Attrition From Premedical Studies Among Latinas: Case Studies. Hispanic Journal of Behavioral Sciences. 2010 Nov 1;32(4):571–85.

22. Andriole DA, Jeffe DB, Hageman HL, Klingensmith ME, McAlister RP, Whelan AJ. Attrition During Graduate Medical Education: Medical School Perspective. Arch Surg. 2008 Dec 15;143(12):1172.

23. AAMC. How to choose the best premed major [Internet]. Association of American Medical Colleges. 1995 [cited 2020 Apr 5]. Available from: https://students-residents.aamc.org/applying-medical-school/article/how-choose-best-premed-major/

24. Sackett PR, Kuncel NR, Arneson JJ, Cooper SR, Waters SD. Does socioeconomic status explain the relationship between admissions tests and post-secondary academic performance? Psychological bulletin. 2009;135(1):1.

25. Liboff AR, Chopp M. Should the premed requirements in physics be changed? American Journal of Physics. 1979 Apr 1;47(4):331–6.

26. Association of American Medical Colleges. Admission Requirements [Internet]. Association of American Medical Colleges. 2019 [cited 2019 Jul 4]. Available from: https://students-residents.aamc.org/applying-medical-school/applying-medical-school-process/medical-school-admission-requirements/admission-requirements/

27. College Board. AP Credit Policy Search [Internet]. College Board. 2019 [cited 2019 Jun 29]. Available from: https://apstudents.collegeboard.org/getting-credit-placement/search-policies

28. Malgwi CA, Howe MA, Burnaby PA. Influences on Students' Choice of College Major. Journal of Education for Business. 2005 May;80(5):275–82.

29. Arulampalam W, Naylor RA, Smith JP. Dropping out of medical school in the UK: explaining the changes over ten years. Medical Education. 2007;41(4):385–94.

30. Association of American Medical Colleges. Financial Aid [Internet]. Association of American Medical Colleges. 2019 [cited 2019 Jun 29]. Available from: https://students-residents.aamc.org/financial-aid/

31. AAMC. Postbaccalaureate Programs [Internet]. AAMC for Students, Applicants, and Residents. 2020 [cited 2020 Oct 6]. Available from: https://students-residents.aamc.org/postbacc/

32. Frazer NB, Twohig MP. The New Social and Behavioral MCAT Requirements: Inspired Innovation, Missed Opportunity, or Both? Peer Review. 2012;14(4):14–17.

33. AAMC. Holistic Review [Internet]. AAMC. 2020 [cited 2020 Oct 6]. Available from: https://www.aamc.org/services/member-capacity-building/holistic-review

34. Witzburg RA, Sondheimer HM. Holistic Review — Shaping the Medical Profession One Applicant at a Time. New England Journal of Medicine. 2013 Apr 25;368(17):1565–7.

35. Bastedo MN, Bowman NA, Glasener KM, Kelly JL. What are We Talking About When We Talk About Holistic Review? Selective College Admissions and its Effects on Low-SES Students. The Journal of Higher Education. 2018 Sep 3;89(5):782–805.

36. AAMC. Roadmap to excellence: Key concepts for evaluating the impact of medical school holistic admissions. Washington, DC: Association of American Medical Colleges; 2013.

37. Perelman School of Medicine. Admissions Competencies [Internet]. 2020 [cited 2020 Oct 6]. Available from: https://www.med.upenn.edu/admissions/admissions.html

38. Boston University School of Medicine. Selection Factors [Internet]. School of Medicine. 2020 [cited 2020 Oct 6]. Available from: http://www.bumc.bu.edu/busm/admissions/typical-interview-day/

39. Stanford University School of Medicine. Eligibility and Recommendations [Internet]. MD Admissions. 2020 [cited 2020 Oct 6]. Available from: http://med.stanford.edu/md-admissions/how-to-apply/academic-requirements.html

Supporting Information

S1 Table. Variable intercorrelations using sub-sample included in the logistic regression model for Milestone 1.

S2 Table. Variable intercorrelations using sub-sample included in the logistic regression model for Milestone 2.

S3 Table. Variable intercorrelations using sub-sample included in the logistic regression model for Milestone 3.

S4 Table. Variable intercorrelations using sub-sample included in the logistic regression model for Milestone 4.

Formatted: Font: Bold

Formatted: Font: Bold

Formatted: Font: Bold

Formatted: Font: Bold

Formatted: Normal

Authors' Response to Editor and Reviewer Comments

Authors' response is not italicized.

Your paper received comments from four reviewers. They all found the theme of the paper important. However, they shared several concerns with the paper. I am particularly concerned the missing issues in the paper as these issues if addressed could improved the quality of the paper's message.

Dear Dr. Borrell,

Thank you for initiating reviews on our paper "The process of attrition in pre-medical studies: A large-scale analysis across 102 school" and giving us the opportunity to revise and resubmit. We appreciated the reviews and constructive comments provided by the reviewers and yourself.

It was encouraging to see that reviewers shared our view in the importance of this work. We had a unique opportunity to quantitatively examined the process through which undergraduate students fulfill prerequisite coursework for medical schools using a large multi-cohort, multi-institution sample. Given the popularity of the informal pre-med major and little prior work focusing on attrition in the early pre-medical study stage, we felt that this work is valuable in adding to the current higher education literature.

In our response to reviewer comments, we have made effort to address every reviewer comment point-by-point and to incorporate the feedback into our revised manuscript. Notably, we substantially restructured our Table 1 to describe the key fulfillment rates in a way that is clearer and also include rates at the intersection of gender and race and at various fulfillment milestones. We also tested additional models that included interaction terms between the various predictors. The results were not tabled given the large number of complex models, but we described key findings. Lastly, we added discussions of the recent shift to holistic evaluation of applicants in medical education and its implications for the ongoing relevance of our results.

A minor detail to note is that while "degree goal" was a predictor included in our original submission because it was a variable that existed in the archival data given to us, and has often been found to be predictive of academic performance in past research. However, upon reexamination, we deemed it irrelevant to the current work. We define our total sample as individuals who have intention of becoming physicians, therefore their "degree goal," or the highest degree they hope to pursue, had very little variance. We excluded it in the revised manuscript, but none of the reported findings changed.

We believe that these edits, along with other minor revisions, have significantly improved our manuscript. Thank you again for your consideration.

Reviewer #1: *This manuscript provides a valuable analysis of the academic trajectories of students who enter college with an interest in pursuing premedical studies, and subsequently to*

attend medical school. Using a large data set provided by the College Board, the authors were able to identify students who had expressed the interest in premedical studies based on students' response to a question administered at the time students took the SAT exam. As the authors indicate, more than 15,000 students indicated the intent to pursue premedical studies once they entered college.

The metric the authors utilize to gauge students' continued level of interest in being premed is the extent to which students complete the science courses that have traditionally been required by medical schools for entry. They analyze the successful completion of these courses in a stepwise manner, as described in the Methods section. Of the students included in the data set, 16.5% completed the full sequence of courses identified by the authors by the time they had completed their undergraduate education. The authors then did an analysis of the academic and demographic factors associated with full completion of the course sequence. They identified gender, race/ethnicity, and prior academic record as among the factors associated with completion of the full course sequence.

This manuscript provides valuable information, potentially useful to those who advise undergraduates in career options. The data set the authors use is unique in its size and the variables it contains. As such, the manuscript adds value to our knowledge of the factors associated with persistence in interest in premedical studies, which is an important factor in the eventual diversity of the physician workforce.

We appreciate the positive feedback on the value of this work. We agree with the reviewer that this dataset provides a unique opportunity to examine the academic trajectories of pre-medical students.

The manuscript reports data on students who entered college between 2006 and 2009. Most of these students would have completed their undergraduate education by 2013-2014. As such, the analysis has missed some fundamental changes that have taken place in the premedical experience. As a result, some of their assumptions and findings may need re-examination by the authors. As one example, the authors state in the Abstract, "Attrition rates are highest initially but drop as students take more relevant courses." The issue is not the relevance of the courses. The issue is how advanced the courses are. The AAMC has done research indicating that inorganic chemistry courses have more relevance to the practice of medicine than do organic chemistry courses. Organic chemistry courses are more advanced than inorganic chemistry courses, but they are not more "relevant".

We have changed "relevant" to "advanced" in the abstract as we agree that later, more advanced courses are not necessarily more relevant that earlier courses. We use the description "relevant" at several other places throughout the manuscript to refer to all courses used to operationalize medical school eligibility that are common prerequisites.

A second thing the authors miss in their discussion is the fundamental shift that has been taking place in the medical school admission process, with the shift to holistic review. In April 2013, Witzburg and Sondheimer published an article in the New England Journal of Medicine titled, "Holistic Review — Shaping the Medical Profession One Applicant at a Time." Under holistic

review, as described by the AAMC, a student's grades in the premedical science courses, while still relevant, are only one of a series of factors used in selecting candidates for medical school. Consistent with the holistic review model, a growing number of medical schools no longer have a specific list of prerequisite courses required for admission. For example, the Perelman School of Medicine at the University of Pennsylvania reports on its Admissions website, "Admissions competencies are not based on specific courses, but rather on the cumulative achievement of knowledge and skills needed to become a physician."
(https://www.med.upenn.edu/admissions/admissions.html)
Similarly, the Admissions webpage of the Stanford University School of Medicine states explicitly, "Stanford Medicine does not have specific course requirements, but recommends appropriate preparation for the study of medicine."
(http://med.stanford.edu/md-admissions/how-to-apply/academic-requirements.html)
Those medical schools that have dropped their explicit course requirements are instead relying on the newly formatted MCAT to provide a metric of scientific and behavioral competencies. Accordingly, in order for this manuscript to sustain its relevance to the current premedical experience, the authors should include a full discussion of the changes that have taken place since their study cohort graduated from college.

We thank the reviewer for pointing out the substantial shift in the evaluation process of medical school applicants. Given when our data was collected and how relatively uniform the prerequisite requirements were across medical schools during that time period, we were able to operationalize medical school eligibility using coursework. As the emphasis on prerequisites relaxes, this approach will become no longer reliable. Per the reviewer's suggestion, we add some language in the limitations and future directions section to highlight this issue.

There is another important factor the authors have left out of their analysis and discussion. While most students who enter college with an intent to pursue premedical studies will complete the sequence of science courses at their undergraduate institution, a substantial number will elect to take either no science courses or some science courses at their undergraduate institution, while completing the full list of traditional science courses as part of a postbaccalaureate premedical program. The AAMC currently identifies 267 programs nationally (https://apps.aamc.org/postbac/#/index) that offer students the opportunity to complete their premedical course sequence after they have graduated from their undergraduate institution. Most of these programs have been active for more than a decade, and thus were available to the students included in the authors' data set. The authors are unable to identify which of the students elected to take only some courses as an undergraduate, while completing the course sequence in a past-bac program.

It is certainly true that our approach of identifying students with intention in studying medicine and operationalizing their continued persistence is not perfect. As the first of the limitations we pointed out, by focusing on coursework, we inevitably include those that have the required coursework for most medical schools but nevertheless lost their intention to study medicine along the way. We thank the reviewer for pointing out another group considered as noise,

namely those that do not complete the required coursework during their undergraduate years, but eventually do in a postbaccalaureate program. We added language to reflect this limitation.

I should point out that, due to poor quality graphics of Figure 1 in the manuscript, I was unable to decipher the figure. The text and images were so faint that I could not read them. I would ask the authors to revise the figure with improved graphics.

The figure has been regenerated to be higher-quality.

As one final issue, I suggest the authors include in their discussion the results of a paper that was published this month that reported on the association between grades in undergraduate chemistry classes and persistence in STEM majors:
R. B. Harris et al. Reducing achievement gaps in undergraduate general chemistry could lift underrepresented students into a "hyperpersistent zone". Science Advances. 10 Jun 2020: Vol. 6, no. 24, eaaz5687 DOI: 10.1126/sciadv.aaz5687

We have added reference of the study. We found the key results of interaction between race and grades for predicting subsequent persistence intriguing. Therefore, we also tested models additional to the ones reported in Table 3 that included various interactions between demographic dummies and continuous predictors. Because this yielded 32 additional models that contained largely redundant and statistically insignificant results, they are not tabled and presented. However, we added a paragraph at the end of the Results section describing the significant interactions.

Reviewer #2: *0. This paper is important for empirically investigating the 'pre-med pipeline,' where many students enter into pre-med (an unspecified 'major' at many schools, including mine) -- and many drop out. Who are these students, and how does dropout unfold over time? That is the focus of the work, which should be of great interest to a wide range of stakeholders: e.g., universities, students and parents, STEM researchers, and medical educators and institutions. Authors have large-scale transcript data across 102 institutions; they have students' major intentions prior to college enrollment -- an impressive data set to answer these questions.*

1. A passing grade (.7 GPA or higher) was used as a screen for eligibility to continue taking pre-med courses, but authors might clarify how this was used (being eligible is necessary but not sufficient for taking the next course). Related to this (but entirely optional), authors might indicate % failure (and didn't retake) of initial courses by demographics as one barrier to entry; and/or investigating course grades more generally might predict staying in pre-med.

As we operationalized medical school eligibility as the fulfillment of various relevant courses, the passing grade was merely used as a minimum standard for which a course can be counted toward achievement. For example, the first milestone requires the completion of one semester of general chemistry, biology, and physics with a passing grade. We revised the language of the four milestones to make this clearer.

2. p. 13 - One can easily appreciate how SAT, HSGPA, and CGPA would be higher for course-fulfillers, to the extent non-fulfilling is driven by obtaining low grades (or failures) in pre-med

courses; this would suggest examining or at least suggesting course grades as an explanatory mechanism (see previous point). Regarding SES differences, could they be higher if college dropouts were included (those who couldn't afford to continue at all, let alone in pre-med)? Dropout might be a significant problem for pre-med and STEM majors, and for URM students, but the current sample does not include this group (maybe include this point somewhere in the discussion section, perhaps with some data on % dropout in the College Board student data).

We agree with the reviewer about the merit of including course grades as a predictor. Our current logistic regression models include average prior relevant course grade as a predictor when possible. We also tested but did not report results of models that included individual course grades separately, as the models currently reported are more parsimonious and yielded similar results.

Unfortunately, the data we were given do not let us identify students that drop out. By examining coursework data, we are able to identify individuals that have coursework up to some point during their undergraduate years, but do not complete all coursework. However, we have no means of distinguishing between those that truly dropped out and those that transferred to a different institution.

3. Table 3 and related text - (a) interpretations of odds ratios need to be refined (they are with respect to the reference group of white females, not overall; they also need to take other predictors into account); (b) odds ratios can be usefully compared against base rate %s from Table 2 and the raw data (e.g., plug in values into the equation to get predicted probabilities, to compare against base rate%s); (c) the two previous points get at whether the pseudo-R2 values are practically useful; but in addition to this, I'd recommend a formal statistical comparison between adjacent models (e.g., compare AIC values).

We thank the reviewer for urging us to report regression findings with more accurate language. We have revised the results section to specify the reference group of the dummy variables as well as the interpretation of coefficients when controlling for all other predictors.

We also agree that odds ratios can be unintuitive and difficult to interpret, and describing the effects in terms of predicted probabilities can facilitate interpretation. Therefore, we have revised the results section by translating some of the key coefficients and odds ratios into differences in likelihood.

We have also added AIC values of the regression models that we tested in Table 3.

4. A bit surprisingly, no institutional differences were provided -- even just adding something such as the distribution of %fulfill by institution would be interesting to readers (and this could be relatively anonymous, given 102 institutions).

We agree with the reviewer that some attention should be paid to institutional differences. The sample sizes per institution varied substantially in the current data, ranging from 2 to 1,475. Therefore, proportions of students that fulfilled coursework milestones in institutions for which we only had sample sizes in the single digits could lead to misleading conclusions. However, we provide an added paragraph near the beginning of the results section with the mean and standard deviation of fulfillment proportion across institutions. We hope this sheds some light on the fulfillment distributions.

Side notes:
5. Table 1 - proportion row/column information was useful, but the labeling was a little confusing

We agree that the original Table 1 could benefit from clearer labels. In addition, we received suggestions from other reviewers to expand this table to also look at the fulfillment breakdown at the intersection of gender and race, as well as to look at fulfillment proportions at the intermediate milestones. In an effort to take into consideration all of these suggestions, we completely restructured our Table 1 to provide the sample size and percentage of individuals that fulfilled each of the four coursework milestones for the entire sample, by gender, by race, and by gender and race. By doing so, we focus the purpose of the table on displaying the proportion who remain med-school-eligible at each stage, and how the proportions compare across demographic groups. We think that this significantly improves the interpretability and readability of the table.

6. Table 2 - (a) the # of courses help define the fulfill/non-fulfill categories so the d-values may not be surprising (if you include them, also check out the distributions of N, as maybe there's positive skew and you consider a more robust d-value involving medians); (c) format the rows a little more descriptively (headers? better labels?)

The reviewer is correct in pointing out that the number of courses taken are quite different between fulfillers and non-fulfillers by definition and they are skewed. We have revised the table to show Cliff's delta rather than Cohen's d for those variables. Row names have been revised to be more descriptive and sub-headings have been added better describe the variables.

Reviewer #3: This study examines a key outcome in the medical education track, and brings to light quantitative research findings that should provide a valuable contribution to the literature for policy-makers and admissions officers alike. The submission is clearly structured, and the analyses are appropriate to the research question, which complements existing work with a more quantitative and large scale data-based approach. I only have a handful of comments.

1) The limitations mentioned in the study are appropriate to mention, but I would have liked to also see further discussion on the relevance of data that is between 11 and 14 years old to the world of medical education today. How stable are the trends that are noted in comparison to the trends of today? Beyond noting the structural changes to MCAT, are there other factors that would lead to caution in drawing strong conclusions from this data, such as the final sentence of the conclusion?

We thank the reviewer for the thoughtful comment. There certainly are additional factors that might influence the generalizability and relevance of the findings. A major one is the endorsement of "holistic review" by medical applicant evaluation and the shift from primary emphasis on academic performance to a consideration of all aspects of applicants' experiences and potential. We added some language in the limitations section explaining this shift in the past decade and its implications.

2) When comparing fulfillers and non-fulfillers of medical school prerequisites, the paper highlights and contextualizes the larger proportion of male fulfillers, but is there a clarifying sentence that can also be added regarding meaningful differences with regards to race/ethnicity?

We have revised the description of those results to reflect the differences.

3) Examining subgroup differences in attrition is a key valuable component of the study, and to that end the paper would be improved by some examination or at the very least mention of interactive effects between race/ethnicity and gender and attrition. For example, the report Altering the Course: Black Males in Medicine highlights the percentage of black male medical school applicants as the lowest among any subgroup. Would conclusions such as ""Lastly, African American students did not differ from White students in their likelihood of persistence at any point." (p 19) differ when differentiating between black men and black women?

We think that the reviewer brings up an important issue regarding the interaction between gender and race for likelihood of persistence. We tried to address this issue in two different ways. First, we expanded and restructured Table 1 to describe not only fulfillment rates by gender and by race separately, but also at the intersection of race and gender. This allows us to compare both the number of individuals who declare some intention to pursue medicine as well as persistence patterns over time across demographic groups (e.g., between Black men and Black women). In addition to these descriptives, we also tested the same four models whose results are displayed in Table 3 when including the interaction terms between gender and race dummy variables. Because the inclusion of interaction terms would add an additional table that is similar to but much larger than Table 3 and more cumbersome to interpret, and most of the coefficients are statistically not significant, we did not table those results but describe the significant effects at the end of the Results section.

Reviewer #4: This study investigates the completion rates of students completing the sequence of undergraduate natural science coursework prerequisite for entry to medical school in the U.S. using a large-scale, multi-institution dataset. It examines the associations of multiple sociodemographic, pre-college achievement, career aspiration, and college-level achievement measures in relation to students' completion of four milestone events defined by natural science coursework from the first semester through advanced organic or biochemistry courses.

The study showed that, of those who reported intending to pursue a medical degree when they took the SAT in high school, only 39% completed the first in a sequence of courses that together provide the foundation needed to be ready for medical school. Completion rates were progressively higher for the (likely) increasingly select group of students achieving each of the three subsequent milestones of coursework. In the end, the overall completion rate of all four

milestones was 16.5%. Observed proportions completing the full sequence were higher for males and Asian students, and lower for females and students from races/ethnicities underrepresented in medicine (i.e., American Indian, Black, and Hispanic).

High school GPA, SAT scores, SES, Gender (Male), and Race (Asian) were associated with greater likelihoods of completing the first set of milestone courses. Gender (Male), Race (Hispanic), and college grades in the first milestone courses were associated with greater likelihoods of completing the second milestone courses, which included the second semester courses of the subjects comprising the first milestone. SAT scores and high school GPA were associated with greater likelihoods of completing the third milestone courses, and race (Hispanic) with a lower likelihood. Finally, gender (Male) and intention to pursue medical school were associated with greater likelihoods of completing the fourth milestone courses, while SAT scores were associated with a lower likelihood of completing the fourth milestone courses.

Although this study has the potential to enhance our understanding of the points at which those interested in the medical profession drop out, the paper fails to make a strong case for it.

1. The authors should consider rewriting the introduction and discussion with a clearer focus and relevant citations, considering the following as suggestions.
• Integrating studies about the factors that contribute to a lack of diversity in STEM or medicine that occur during or before college with the present study, including those referenced in the current about "the leaky pipeline." References 4, and 11-19 seem germane to the study's focus.
• Addressing, alongside things like gender norms that might affect persistence, comparable treatment of the lower rates of completion for those from racial/ethnic backgrounds underrepresented in medicine who, more often have lower-quality middle and high school education. Addressing the role that high school preparation might play in students first-semester performance in natural science coursework and beyond would strengthen the paper.
• Removing or clarifying the relevance of literature on burnout/risk of attrition in medical school (references 3, and 5-9), and references 3, 10, and 20, which about attrition in the medical school in the U.K., which differs substantially from medical school in the U.S. The authors also should consider eliminating references about attrition in U.S. medical school (given that 95% of medical students graduate within five years) or make a stronger case for their relevance.

We have revised the introduction and discussion following some of the reviewers' suggestions, including removing references of studies of medical school in UK, removing review of the literature on attrition in medical school, and adding some language on systemic underrepresentation of ethnic minority groups in medicine.

2. With a clearer focus, the authors might present their results differently to highlight important findings. For example, Table 1 shows that Black and Hispanic students completed all four milestones at lower rates than White or Asian students. It might be important, given the research questions, to know at which milestone(s) they did not progress, in addition to showing their prevalence at the two end points. Similarly, it was confusing on page 19 to read that African

American students did not differ from White students in their likelihood of persistence at any point, even though only 9% of Black students completed all four milestones, compared to 16% of White students. The authors should address how the observed and predicted rates of completion lead to different interpretations.

3. Similar suggestions would improve the discussion of the paper's findings.

We have expanded and restructured Table 1 and present fulfillment rates by racial subgroups at various milestones, following the reviewer's suggestions. We agree that by doing so, we gain a more complete understanding of the process of attrition.

We thank the reviewer for pointing out the confusion in presenting proportion of fulfillment by group and including those subgroup dummy variables in the regression models. For example, the African American group had the overall lowest proportion of overall eligibility based on their coursework. However, this difference seems to go away in the predictive models once the other predictors (e.g., SES, SAT scores, and grades) are controlled. We have revised the language in the Results and Discussion sections to clarify this.

4. Finally, the conclusions are unfounded and should be rewritten.

5. Other minor suggestions include:
• addressing how the increasingly select sample of students might affect the results of the logistic regression analyses. For example, SES, SAT scores, and high school GPA may be restricted as the sample reduces from more than 13,000 to about 2,500 students.

• Replacing outdated references with more recent research (e.g., 18) and confirming the appropriateness of journals cited (e.g., 9, which is missing the Journal Name "Journal of Unschooling and Alternative Learning").

• Clarifying the relevance or eliminating the text describing challenges identifying premed majors. The study documented the process for identifying courses prerequisite for medical school (and that are well described on each medical school's website).

References have been edited and the Conclusion section was rewritten.
